# Intelligent Manufacturing for Osteoarthritis Organoids

**DOI:** 10.1111/cpr.70043

**Published:** 2025-04-26

**Authors:** Xukun Lyu, Jian Wang, Jiacan Su

**Affiliations:** ^1^ Department of Orthopedics Xinhua Hospital Affiliated to Shanghai Jiao Tong University School of Medicine Shanghai China; ^2^ Trauma Orthopedics Center Xinhua Hospital Affiliated to Shanghai Jiao Tong University School of Medicine Shanghai China; ^3^ Institute of Musculoskeletal Injury and Translational Medicine of Organoids Xinhua Hospital Affiliated to Shanghai Jiao Tong University School of Medicine Shanghai China; ^4^ Department of Clinical Medicine Shanghai Jiao Tong University School of Medicine Shanghai China; ^5^ Institute of Translational Medicine Shanghai University Shanghai China; ^6^ National Center for Translational Medicine SHU Branch Shanghai University Shanghai China

**Keywords:** arthrosis, artificial intelligence, cartilage, in vitro modelling, intelligent manufacturing, osteoarthritis organoids

## Abstract

Osteoarthritis (OA) is the most prevalent degenerative joint disease worldwide, imposing a substantial global disease burden. However, its pathogenesis remains incompletely understood, and effective treatment strategies are still lacking. Organoid technology, in which stem cells or progenitor cells self‐organise into miniature tissue structures under three‐dimensional (3D) culture conditions, provides a promising in vitro platform for simulating the pathological microenvironment of OA. This approach can be employed to investigate disease mechanisms, carry out high‐throughput drug screening and facilitate personalised therapies. This review summarises joint structure, OA pathogenesis and pathological manifestations, thereby establishing the disease context for the application of organoid technology. It then examines the components of the arthrosis organoid system, specifically addressing cartilage, subchondral bone, synovium, skeletal muscle and ligament organoids. Furthermore, it details various strategies for constructing OA organoids, including considerations of cell selection, pathological classification and fabrication techniques. Notably, this review introduces the concept of intelligent manufacturing of OA organoids by incorporating emerging engineering technologies such as artificial intelligence (AI) into the organoid fabrication process, thereby forming an innovative software and hardware cluster. Lastly, this review discusses the challenges currently facing intelligent OA organoid manufacturing and highlights future directions for this rapidly evolving field. By offering a comprehensive overview of state‐of‐the‐art methodologies and challenges, this review anticipates that intelligent, automated fabrication of OA organoids will expedite fundamental research, drug discovery and personalised translational applications in the orthopaedic field.

## Graphical abstract

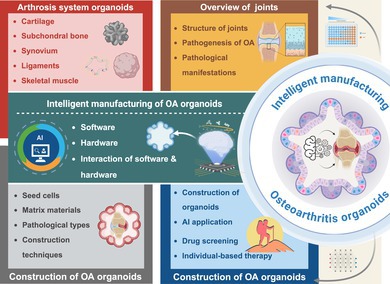

**Abbreviations**
2D: two‐dimensional
3D: three‐dimensional
AAV: adeno‐associated viruses
ADAMTS: a disintegrin and metalloproteinase with thrombospondin motifs
AI: artificial intelligence
AlgMA: alginate methacrylate
BMSCs: bone marrow stromal cells
DIO2: type II iodothyronine deiodinase
DL: deep learning
DLP: digital light processing
DMSO: dimethyl sulfoxide
ECM: extracellular matrix
ECs: endothelial cells
EPCs: endothelial progenitor cells
EpPs: lymphoid progenitor cells
ESCs: embryonic stem cells
GAGs: glycosaminoglycans
GANs: generative adversarial networks
GDF5: genome‐wide association studies
GelMA: methacrylate
GWAS: genome‐wide association studies
HA: hyaluronic acid
HAP: hydroxyapatite
HSCs: haematopoietic stem cells
IL‐1β: interleukin‐1β
IL‐6: interleukin‐6
iPSCs: induced pluripotent stem cells
LSFM: light‐sheet fluorescence microscopy
MkPs: myeloid progenitor cells
ML: machine learning
MMP13: matrix metalloproteinase 13
MPM: multi‐photon microscopy
MSCs: mesenchymal stem cells
NLP: natural language processing
OA: osteoarthritis
OBs: osteoblasts
OCs: osteoclasts
O‐GPT: Organoid‐GPT
PEG: polyethylene glycol
PGs: proteoglycans
PLGA: poly(lactic‐*co*‐glycolic acid)
PSCs: pluripotent stem cells
RA: rheumatoid arthritis
RANKL: nuclear factor‐κ B ligand
RGD: arginine‐glycine‐aspartic acid
rhGH: recombinant human growth hormone
RSD‐MS: RGD‐silk fibroin‐DNA hydrogel microspheres
SCs: satellite cells
sgRNAs: single guide RNAs
TNF‐α: tumour necrosis factor‐α

## 1 Introduction

Osteoarthritis (OA) is the most common degenerative joint disease worldwide, characterised by degenerative changes in articular cartilage and secondary bone hyperplasia. The primary clinical manifestations include joint pain, stiffness, swelling, limited mobility, deformity and potential disability [1]. OA predominantly affects middle‐aged and elderly individuals, with higher prevalence in women [2]. Its disease burden is escalating with aging populations, affecting over 595 million globally [3], including 130 million in China [4]. Key risk factors include aging, sex, genetics, mechanical loading and inflammation [5]. Current treatments, such as weight management, orthotics, analgesics and joint replacement, lack effective strategies targeting cartilage degeneration [6]. The main challenge lies in the incomplete understanding of OA pathogenesis and the absence of reliable in vitro models. Organoid technology has emerged as a promising innovative approach, providing a robust in vitro platform for investigating OA pathogenesis and developing novel therapeutic interventions.

Organoids are miniature tissue structures formed through the self‐organisation of stem cells or progenitor cells specific to three‐dimensional (3D) culture conditions [7, 8]. They can mimic the cellular composition, spatial configuration and partial functional characteristics of native organs. The core principle of organoid formation lies in leveraging the pluripotency and self‐organising ability of stem cells to reconstruct the dynamic microenvironment of organ development or pathological processes in vitro [9, 10]. Significant advancements in organoid technology have been achieved across various tissues, including retinal [11], liver [12, 13], kidney [14], pancreatic [15] and brain organoids [16]. Compared with traditional two‐dimensional (2D) cultures and animal models, organoids better mimic the 3D structure, cell–cell interactions and ECM composition of their native tissues under both physiological and pathological conditions [17, 18]. Among these, arthrosis organoids represent a rapidly growing field, emerging as a novel platform to model the pathological state of OA patients more accurately. By closely simulating the in vivo disease microenvironment, OA organoids offer a stable in vitro model for studying OA pathology. Due to their ability to faithfully replicate pathological conditions, OA organoids hold great potential as powerful tools for mechanistic studies, drug screening and regenerative medicine research. This innovation is expected to accelerate the development and clinical translation of new OA therapies, addressing the current challenges in OA treatment [19, 20, 21].

**FIGURE 1:**
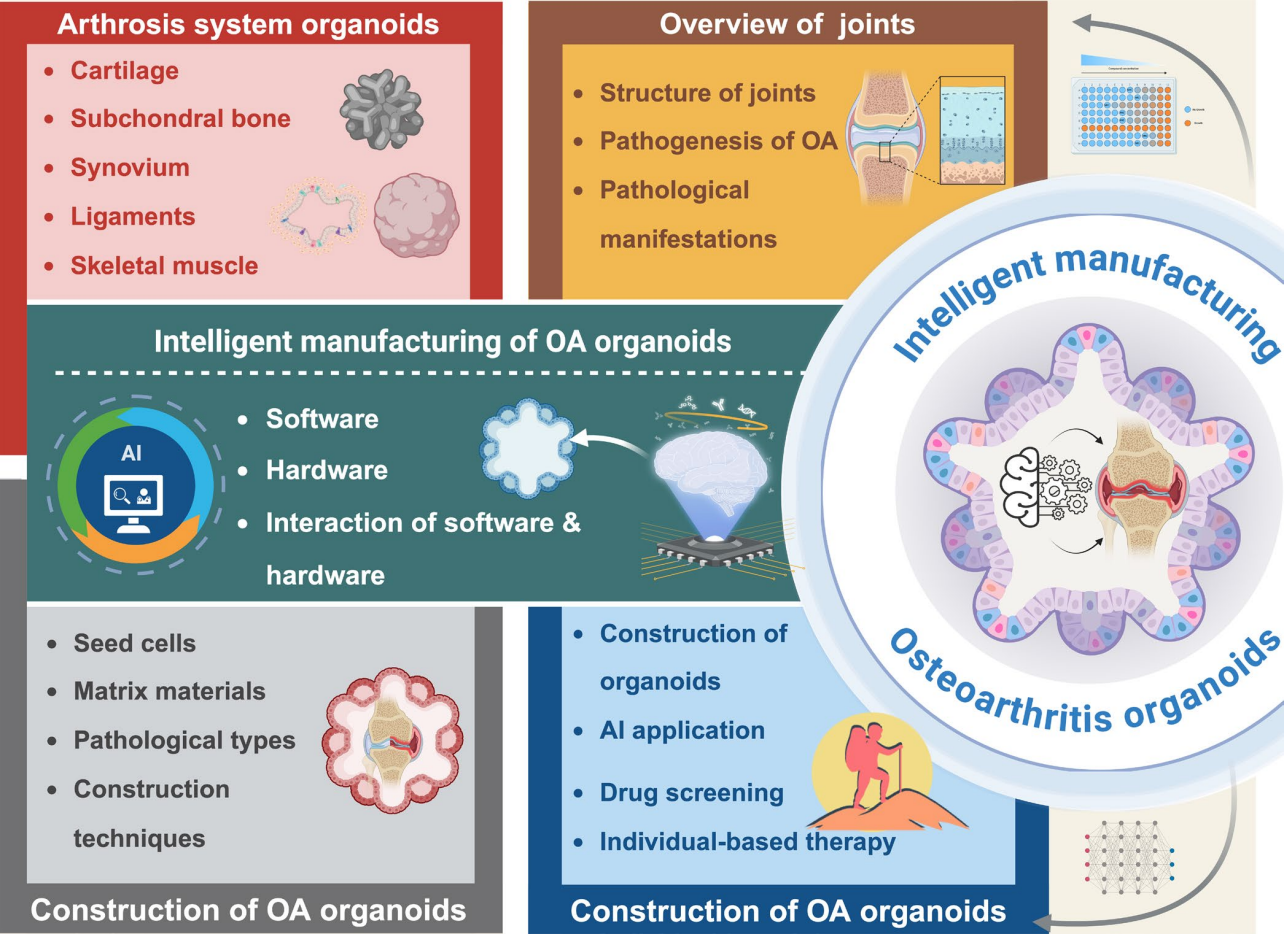
Overall schematic of intelligent manufacturing in osteoarthritis organoids. Broad overview of the article's structure, outlining the progression from fundamental joint anatomy and OA pathogenesis to organoid construction and, ultimately, to intelligent manufacturing and future prospects. Created with BioRender.com.

This review provides a comprehensive overview of the current pathophysiological research on OA. Following a progressive approach from physiological to pathological models, this review systematically outlines the methodologies for OA pathological organoid fabrication. A key focus of this review is the integration of emerging technologies from computer science, bioengineering and biomaterials science into OA organoid fabrication techniques. This multidisciplinary approach forms the foundation of an intelligent manufacturing framework for OA organoids, referred to as the intelligent manufacturing of OA pathological organoids: a ‘human‐free factory’ approach. By introducing this novel automated, scalable and intelligent OA organoid production method, this review aims to enhance researchers' understanding of OA pathophysiology and facilitate advancements in OA mechanistic studies, therapeutic development and clinical translation (Figure 1).

## 2 Overview of Joints

### 2.1 Joint Structure

The anatomical connection between bone and cartilage is established through fibrous connective tissue, cartilage or osseous tissue, forming skeletal junctions that can be classified into two primary categories: direct and indirect connections. The indirect connection, known as the synovial joint or diarthrosis, represents the most advanced form of skeletal articulation. This structure features a fluid‐filled cavity separating the opposing bone surfaces, connected solely by surrounding connective tissue, thereby providing significant mobility [22]. The fundamental structure of synovial joints comprises three essential components: the articular surface, joint capsule and joint cavity. The articular cartilage, predominantly composed of hyaline cartilage with some fibrocartilage components, covers the articulating bone surfaces. This specialised tissue serves crucial biomechanical functions in shock absorption and lubrication, enabling it to withstand compressive forces and frictional stresses during movement. The structural and functional integrity of cartilage is maintained by its principal constituents: water, collagen fibres and glycosaminoglycans. The joint capsule consists of an outer fibrous membrane and an inner synovial membrane. The synovial membrane secretes synovial fluid, which provides essential lubrication, nutrient supply and friction reduction for joint protection. In addition to these fundamental structures, certain joints develop specialised auxiliary components such as ligaments, articular discs and labra to enhance either flexibility or stability, adapting to specific functional requirements [23]. However, with advancing age or due to traumatic injuries, the physiological architecture of synovial joints may undergo degenerative changes. These alterations typically manifest as cartilage degradation and reduced synovial fluid production, potentially leading to pathological conditions such as OA [24, 25].

### 2.2 Pathogenesis of Osteoarthritis

OA is a chronic joint disease characterised by degenerative changes in articular cartilage and secondary osteophyte formation [26]. Its pathogenesis is complex and involves multiple factors, including inflammatory responses, mechanical stress damage and genetic predisposition [5]. The disease affects articular cartilage as well as other joint tissues, including subchondral bone, synovium and joint capsule. OA commonly occurs in weight‐bearing joints, such as the knee, hip, spine and distal interphalangeal joints [27].

The development of OA is a long‐term, chronic and progressive pathological process [26]. It is generally believed to result from the interaction of multiple pathogenic factors, including both mechanical and biological elements. The primary etiological factors include age, gender, genetic predisposition, mechanical loading, inflammatory responses and obesity [28]. Age is a well‐established risk factor. As individuals age, cartilage matrix degradation increases, chondrocyte apoptosis rises and regenerative capacity declines, making middle‐aged and elderly populations more susceptible to the disease [29]. In terms of gender, the incidence of OA is higher in women than in men, with a notable increase after menopause. This may be related to decreased oestrogen levels, alterations in oestrogen receptors in articular cartilage and enhanced inflammatory responses [30]. OA exhibits a strong genetic predisposition. Twin studies and genome‐wide association studies (GWAS) have shown that the heritability of finger and spinal OA is relatively high, while the genetic correlation of knee OA is comparatively weaker. GWAS studies have identified several OA‐related risk genes, including growth differentiation factor 5 (GDF5), type II iodothyronine deiodinase (DIO2) and matrix metalloproteinase 13 (MMP13). These genes are involved in various pathways, such as chondrocyte proliferation, matrix degradation and inflammation regulation [31, 32]. Mechanical loading and joint biomechanical changes are critical factors in OA development. Abnormal mechanical joint loading (e.g., prolonged knee valgus/varus), joint trauma (e.g., anterior cruciate ligament tears, meniscus injuries) and subchondral bone structural abnormalities (e.g., bone marrow oedema lesions) are closely associated with OA progression [33]. Obesity not only increases the load on the knee and hip joints but also mediates chronic low‐grade inflammation through adipokines such as leptin and adiponectin, accelerating cartilage degradation and subchondral bone remodelling [34].

The pathogenesis of OA can be divided into two main categories: primary and secondary [35]. Primary OA is more common in individuals over 50 years old, with no clear systemic or local predisposing factors, although genetic factors may play a role. Secondary OA is more prevalent in younger adults and can result from trauma, long‐term mechanical stress, inflammatory responses or joint instability. Examples include intra‐articular fractures, joint capsule or ligament laxity causing joint instability and joint deformities such as genu varum or valgus, which alter joint mechanics and lead to OA on the basis of pre‐existing conditions [35]. The most significant pathological changes in OA include deformation, wear and loss of articular cartilage; subchondral bone sclerosis or cystic changes; osteophyte formation at joint margins; synovial hyperplasia; thickening and contracture of the joint capsule and surrounding ligaments; atrophy of periarticular muscles; and ultimately, complete destruction of the joint surface, resulting in joint deformity [36].

### 2.3 Pathological Features of Osteoarthritis

#### 2.3.1 Degeneration of Cartilage

Articular cartilage is the most critical component of synovial joints, consisting of cells and extracellular matrix (ECM) components [37]. The only cell type present in articular cartilage is the chondrocyte, which is embedded within the ECM, maintaining a low metabolic state. Chondrocytes possess primary cilia and other mechanosensitive receptors on their surface, enabling them to sense and adapt to physical forces, thereby strictly regulating the biochemical composition and structural organisation of articular cartilage [38, 39]. The ECM is composed of water (> 70%) and organic extracellular matrix. The organic extracellular matrix forms a stable network structure primarily composed of type II collagen and several other collagens and non‐collagenous proteins, ensuring the tensile strength of cartilage [37, 40]. Various charged proteoglycans embedded in this network structure attract water into the cartilage through their hydrophilic side chains, providing compressive resistance [41].

Articular cartilage is susceptible to external damage, long‐term mechanical stimulation, aging and inflammatory responses, all of which contribute to the development and progression of OA [42]. The degenerative changes in articular cartilage and abnormalities in chondrocytes are the core pathological features of OA, primarily characterised by cartilage matrix degradation, chondrocyte apoptosis and cartilage surface damage. In the early stages of OA, chondrocytes exhibit increased synthetic activity, compensatorily secreting type II collagen and proteoglycans (PGs) to repair the damaged matrix [43]. However, as the disease progresses, the activity of MMPs increases, such as MMP‐1, MMP‐3 and MMP‐13, leading to the degradation of type II collagen and the destruction of the pericellular matrix of chondrocytes, resulting in functional inhibition [44]. Simultaneously, a disintegrin and metalloproteinase with thrombospondin motifs (ADAMTS)‐4/5 promotes cartilage degradation by cleaving aggrecan core proteins and other matrix metalloproteinases [45]. The cartilage loses its hydration capacity, becoming extremely fragile. The depletion of proteoglycans and erosion of the collagen network mark irreversible pathological progression. In OA, the inflammatory response of chondrocytes is significantly enhanced, characterised by increased production of cytokines, chemokines and other pro‐inflammatory substances, including interleukin‐1β (IL‐1β), interleukin‐6 (IL‐6) and tumour necrosis factor (TNF‐α) [46]. Additionally, the autophagy mechanism of chondrocytes declines, leading to increased cell death [47]. As OA progresses, many chondrocytes show increased expression of genes and production of proteins associated with hypertrophy. Angiogenic factors such as vascular endothelial growth factor are also induced, promoting vascular invasion and the expansion of calcified cartilage [48, 49, 50]. Changes in cartilage composition lead to significant alterations in its material properties, increasing susceptibility to physical damage and further exacerbating chondrocyte dysfunction and cartilage surface damage [37]. In terms of gross pathological changes, early stage OA patients exhibit surface fibrillation of articular cartilage, which becomes yellowish and loses its lustre. This is followed by localised softening and loss of elasticity [51]. During weight‐bearing activities, the cartilage surface becomes rough and undergoes fragmented peeling. Deep fissures in the articular cartilage and the formation of intra‐articular loose bodies eventually lead to cartilage shedding, exposure of the underlying calcified cartilage and subchondral bone [52].

#### 2.3.2 Subchondral Bone Remodelling

The anatomical composition of subchondral bone includes the subchondral bone plate (cortical plate) and subchondral trabecular bone, forming the tidemark as the mechanical interface with cartilage. Subchondral bone undergoes complex, dynamic structural remodelling and molecular regulation imbalances in OA, with pathological changes involving anatomical abnormalities, cellular dynamic imbalances and mechanical conduction disturbances [53, 54]. The subchondral bone cortical plate thickens and bone mineralisation status changes. Weinans et al. noted that although subchondral bone volume increases in OA, bone hardness decreases due to reduced bone mineral density [55]. Trabecular bone undergoes remodelling, with decreased trabecular thickness, increased spacing and microcrack formation. Gallant et al. indicated that these trabecular changes are directly related to increased mechanical load [56]. Additionally, characteristic anatomical pathological markers form in subchondral bone, including subchondral cysts resulting from osteoclast‐mediated bone resorption and fluid accumulation, as well as marginal osteophytes formed through endochondral ossification [57]. Pelletier et al.'s MRI imaging studies suggested that increased subchondral cyst size correlates with cartilage volume loss in the same region, highlighting the importance of subchondral bone lesions in OA pathophysiology [58]. Donald et al. conducted MRI imaging studies showing that bone marrow lesions and micro‐fractures coexist in subchondral bone, indicating an imbalance in the dynamic equilibrium between local repair and destruction [59]. Abnormal bone remodelling in subchondral bone is driven by functional imbalances among osteoblasts (OBs), osteoclasts (OCs) and endothelial cells (ECs) [54]. Su et al. identified three OBs subtypes involved in abnormal subchondral bone remodelling through single‐cell sequencing: endothelial osteoblasts (promoting angiogenesis via VEGF signalling), stromal osteoblasts (leading collagen fibre assembly) and mineralising osteoblasts (mediating abnormal mineralisation as terminally differentiated cells, leading to increased bone fragility) [60]. The vascular‐osteogenic coupling mechanism also promotes abnormal subchondral bone remodelling [61]. Shiwu et al. found that pre‐endothelial cells form functional coupling with OBs through the TGFβ/VEGF/NOTCH pathway [54]. The differentiated H‐type ECs not only promote pathological vascular invasion but also activate osteoclasts by releasing receptor activator of nuclear factor‐κ B ligand (RANKL) [62], further recruiting bone progenitor cells through the Smad2/3 pathway to accelerate abnormal bone turnover, forming a ‘vascular‐osteogenic‐osteoclastic’ vicious cycle. This cellular network dysregulation leads to abnormal bone turnover rates, manifested as osteoid island deposition and osteophyte formation [63]. In the early stage, abnormal vascularisation dominates. Christelle et al. noted that increased mechanical load triggers OBs to secrete inflammatory mediators such as IL‐6 and MMP‐13 [64], while H‐type vessels penetrate the tidemark, with MRI revealing bone marrow lesions and micro‐fractures [59]. In the middle stage, characteristic subchondral cysts form. Michel et al. indicated that their formation mechanism is related to osteoclast‐mediated bone resorption and fluid accumulation, with reduced cortical plate hardness exacerbating cartilage deformation [65]. In the late stage, marginal osteophyte proliferation occurs through endochondral ossification. Although this provides compensatory joint stability, it alters joint mechanical distribution [66, 67]. Notably, Felson et al. pointed out that osteophyte formation is closely related to pain, possibly due to the rich innervation of subchondral bone [67]. Studies have shown that both bone sclerosis and softening accelerate cartilage degeneration by altering stress distribution [68, 69]. The molecular basis of bone–cartilage interaction provides new therapeutic targets for OA, such as anti‐angiogenic therapy targeting H‐type vessels, interventions in Pre‐ECs differentiation to block pathological bone remodelling or epigenetic strategies to regulate OBs differentiation. However, current research still faces technical limitations, such as the loss of osteoclast phenotypes during single‐cell isolation, necessitating methodological breakthroughs [60].

#### 2.3.3 Synovial Inflammatory Hyperplasia

**FIGURE 2:**
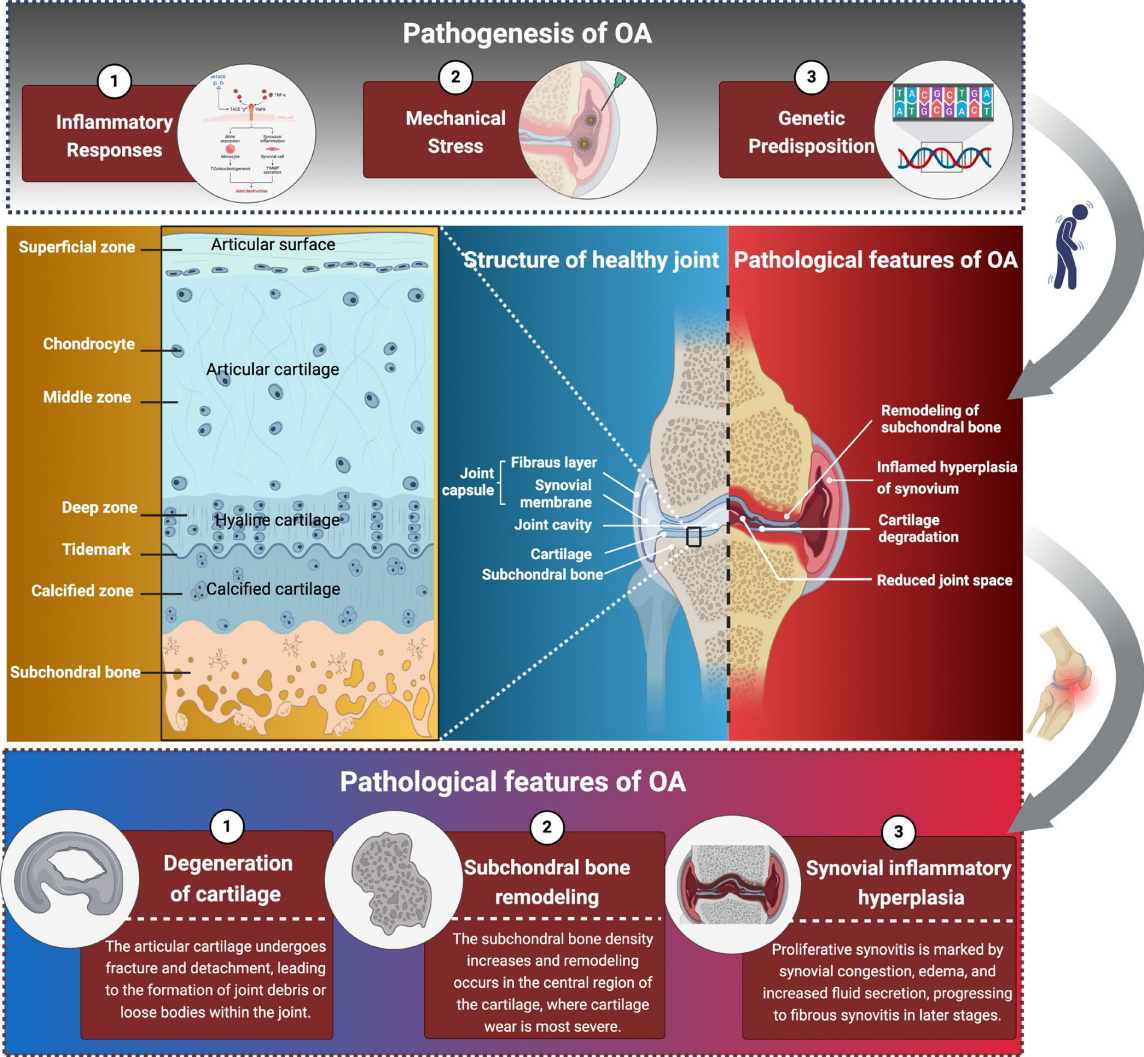
Comparative depiction of healthy joint anatomy and osteoarthritis pathology. Comparing healthy joints and OA joints, highlighting key pathological changes such as cartilage degeneration, synovial inflammation and bone remodelling. Created with BioRender.com.

The synovial membrane is located on the inner layer of the joint capsule, covering the ligaments and tendons within the joint. It consists of an outer layer of synovial epithelial cells and an inner layer of synovial fibroblasts. Physiologically, the cellular components of the synovial membrane secrete synovial fluid, which contains a high concentration of hyaluronic acid, playing a crucial role in lubrication during joint movement. During the pathological progression of OA, significant structural and functional alterations occur in the synovium, which are closely associated with symptoms such as pain and synovitis. Synovial inflammation represents a critical aspect of OA progression, characterised by pathological features including fibrosis, macrophage infiltration and synovial epithelial hyperplasia [70]. Synovial inflammation exhibits distinct pathological characteristics at different stages of OA. In early‐stage OA, synovial inflammation typically manifests as mild hyperplasia and macrophage infiltration. As the disease progresses, synovial inflammation intensifies, presenting with pronounced fibrosis and synovial hyperplasia [71]. This inflammatory response is not confined to the synovial layer but may also affect sub‐synovial tissues, leading to the accumulation of local immune cells and the release of pro‐inflammatory factors [72]. Synovial fibrosis is one of the key pathological features of synovial inflammation in OA. Research indicates that synovial fibroblasts (SFs) play a significant role in OA by secreting collagen and MMPs, thereby promoting cartilage degradation and synovial fibrosis [73]. Additionally, macrophages and T cells within the synovium contribute to the inflammatory response by releasing pro‐inflammatory factors such as IL‐6 and TNF‐α, further exacerbating synovial pathological changes [74] (Figure 2).

## 3 Arthrosis System Organoids

The key characteristics of arthrosis organoids are structural biomimicry and functional dynamics. Compared with traditional 2D in vitro models, arthrosis organoids better simulate the structural complexity, functionality and cellular diversity of in vivo joints. Additionally, they can recapitulate essential functional characteristics such as mechanical sensing and response, simulation of the synovial membrane and synovial fluid and subchondral bone mineralisation and remodelling. The construction of arthrosis organoids is a multi‐step, integrative process involving various biomaterials. Establishing effective construction methods requires a profound understanding of the physiological and pathological mechanisms of joints, as well as the biological, physical and chemical factors influencing the development of intra‐articular structures such as bone and cartilage [75, 76]. Given the complexity of joint anatomy and physiology, the construction of arthrosis organoids integrates multiple organoid structures, primarily including cartilage organoids, subchondral bone organoids, synovial organoids, skeletal muscle organoids and ligament organoids, which are subsequently assembled in vitro into complete arthrosis organoids. The construction process necessitates appropriate cell sources, biomaterial scaffolds, differentiation induction strategies and biofabrication techniques. It follows strict protocols that are continuously optimised based on organoid growth conditions to ensure maturation, aiming to replicate the intricate structure and physiological function of native joints as accurately as possible [77].

**FIGURE 3:**
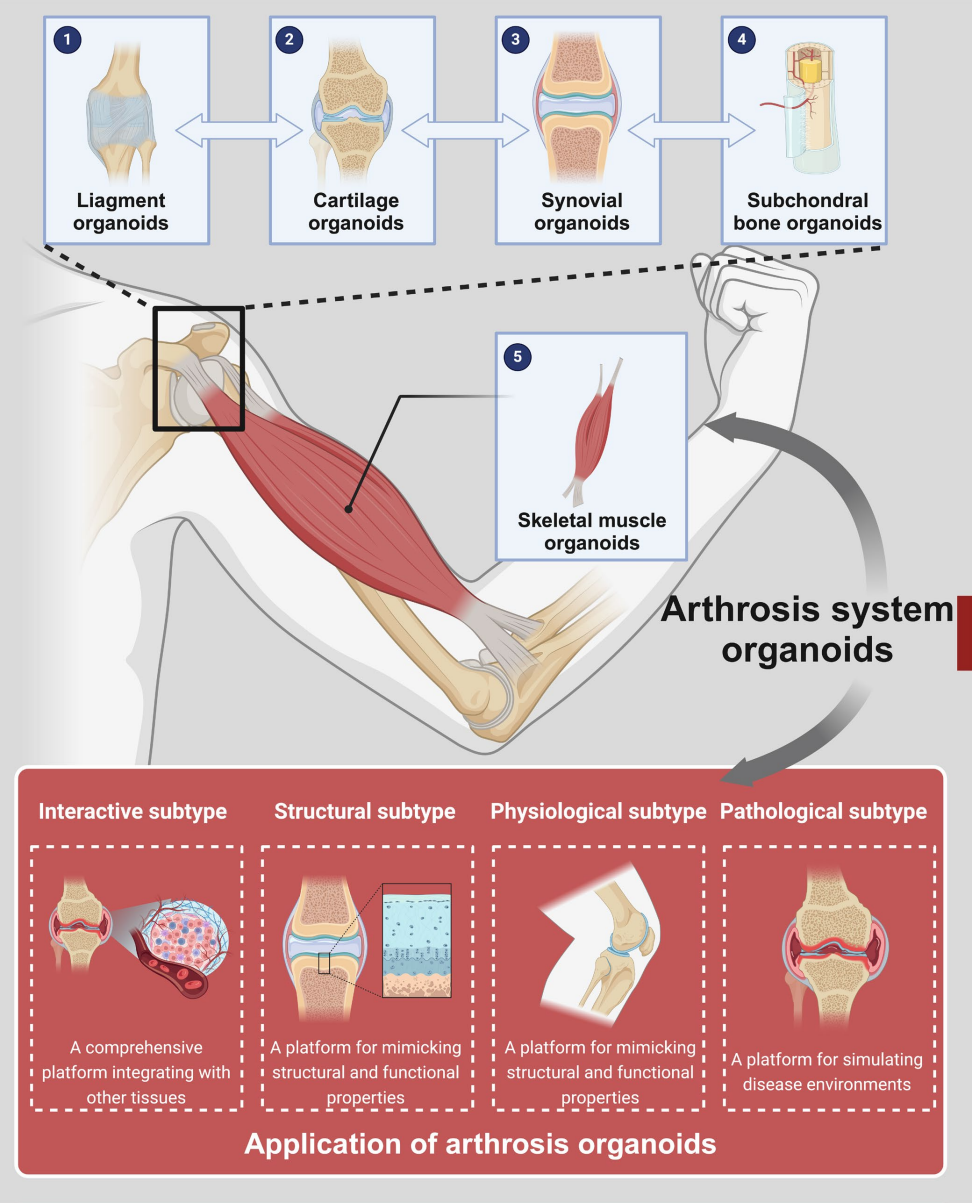
Subtypes of joint organoids and their application scenarios. Presenting subtypes of organoids, including cartilage, synovium, ligament and bone, and their applications in basic research and disease modelling. Created with BioRender.com.

The applications of arthrosis organoids can be categorised into the following four types: physiological subtype: a platform for simulating growth and development; pathological subtype: a platform for simulating disease environments; structural subtype: a platform for mimicking structural and functional properties; and interactive subtype: a comprehensive platform integrating with other tissues. researchers can leverage these four arthrosis organoid subtypes for multi‐dimensional studies, facilitating the in vitro simulation of key pathological features such as cartilage degradation, synovial inflammation, mechanical stress injuries and genetic influences. These studies will advance our understanding of disease pathogenesis, identify novel therapeutic targets and enable high‐throughput drug screening, ultimately driving the progress of regenerative medicine and precision medicine [24] (Figure 3).

### 3.1 Cartilage Organoids

The construction strategies for cartilage organoids exhibit increasing diversity, with a core focus on mimicking the ECM characteristics and mechanical environment of natural cartilage. From the perspective of seed cells, cartilage organoids are primarily derived from embryonic stem cells (ESCs) [78], induced pluripotent stem cells (iPSCs) [79], mesenchymal stem cells (MSCs) [80] and human periosteum‐derived cells [81], all of which have been successfully induced to differentiate into cartilage organoids in vitro or in vivo. Traditional cartilage organoid construction mainly relies on in vitro culture techniques, utilising natural biomaterials such as collagen and hyaluronic acid as scaffolds, along with autologous chondrocyte or seed cell implantation [82]. Lin et al. developed a cartilage organoid model using a directed chondrogenic progenitor cell induction strategy, enabling dynamic simulation of cartilage homeostasis and degenerative processes [83]. Li et al., based on MSCs, utilised verteporfin to regulate YAP and employed decellularised matrix scaffolds as the support, successfully constructing a cartilage organoid that simulates cartilage development [84]. Although these early methods were widely adopted, they presented several limitations, such as low cell proliferation and differentiation efficiency, insufficient mechanical properties of scaffolds and the inability to replicate the complex structure of natural cartilage [85]. In recent years, advancements in 3D printing, bioprinting and multilayer scaffold technologies have led to more diverse and precise strategies for cartilage organoid construction. The 3D printing technology enables the fabrication of multi‐layered, porous scaffolds, allowing for high‐precision construction of complex cartilage structures while promoting chondrocyte proliferation and differentiation [86]. Ouyang et al. developed a rapid bone regeneration strategy using 3D printing, demonstrating effective cartilage differentiation in vitro and successful cartilage repair after implantation in vivo [87]. Similarly, Guo et al. combined bioactive molecules (e.g., GDF‐5) with 3D printing technology to construct hierarchical scaffolds that simulate the complexity of natural cartilage. This strategy not only enhanced scaffold mechanical properties but also significantly improved Bone marrow stromal cells (BMSCs) migration and chondrogenic differentiation, facilitating joint cartilage regeneration [88]. Traditional materials used for cartilage organoid construction include natural biomaterials such as collagen and hyaluronic acid, as well as synthetic polymers like polylactic acid and polyhydroxyalkanoates. However, these materials often suffer from low biocompatibility, immune interference and poor mechanical support [89]. Recent breakthroughs in novel biomaterials, such as advanced hydrogels and multilayer composite materials, have significantly improved cartilage organoid construction. High‐density culture and microenvironment simulation technologies are essential for cartilage organoid development. For instance, GelMA microspheres or 3D scaffolds combined with TGF‐β3 induction can drive MSCs toward functional chondrocyte differentiation and layered cartilage formation [90]. Su's team was the first to integrate DNA with silk fibroin to create a dual‐network DNA‐silk fibroin hydrogel with tunable surface stiffness, effectively regulating stem cell chondrogenic differentiation [91]. Furthermore, they introduced photo‐crosslinking and self‐assembly techniques into a microfluidic‐integrated system, developing novel RSD‐MS. This system upregulated integrin‐mediated cell adhesion and localised adhesion pathways, promoting glycosaminoglycan biosynthesis and BMSC chondrogenic differentiation, thereby establishing a stable platform for cartilage organoid construction based on composite biomaterials [92].

The applications of cartilage organoids primarily focus on their physiological and pathological subtypes. Physiological cartilage organoids offer novel solutions for cartilage regeneration and repair. By implanting cartilage organoids into damaged regions, they can accelerate the repair process by providing mechanical support and releasing growth factors. Su et al. developed cartilage organoids with an optimal surface stiffness dual‐network DNA‐silk fibroin hydrogel, which upregulated the Wnt and TGF‐β signalling pathways in BMSCs, promoting collagen‐containing extracellular matrix secretion and accelerating the repair of articular cartilage defects [91]. Pathological cartilage organoids are widely utilised for modelling human cartilage‐related diseases, such as OA and cartilage injuries. These models allow researchers to study chondrocyte differentiation dynamics, signalling pathways and drug screening. Additionally, they serve as platforms for evaluating personalised regenerative therapies, employing transcriptomics, epigenetics and metabolomics to analyse cartilage tissue characteristics [83]. Dönges et al. successfully established a cartilage organoid system based on BMSCs, replicating key pathological features of OA, including pathological cartilage hypertrophy, aberrant extracellular matrix mineralisation, activation of catabolic pathways and increased tissue mechanical stiffness [93]. Similarly, Abe et al. developed a human iPSCs‐derived cartilage organoid model, which revealed that a pathogenic missense mutation in a familial early‐onset OA cohort significantly weakened the binding affinity between fibronectin and type II collagen. This impairment disrupted cartilage formation and accelerated the pathological progression of OA [94].

### 3.2 Subchondral Bone Organoids

Subchondral bone organoids constitute an essential component of bone organoids. Bone organoids are 3D biological tissue models that simulate the natural developmental processes of bone, recapitulating its structural and functional characteristics. These organoids are typically composed of stem cell‐derived differentiated cells combined with biomaterials, enabling the formation of early bone tissue through mechanical stimulation or the influence of inductive factors [95]. The construction of bone organoids relies on stem cell technologies, biomaterials and fabrication techniques. Using MSCs, iPSCs and BMSCs as cellular sources, bone organoids can be constructed with the support of natural or synthetic biomaterials. The development of these models is facilitated by 3D printing, scaffold‐free self‐organisation techniques and bioreactor‐based approaches, leading to the formation of bone organoids containing bone marrow‐derived MSCs, haematopoietic cells, fibroblasts and adipose tissue [24]. Traditional classifications of bone organoids include osteo‐callus organoids, woven bone organoids, trabecular bone organoids and bone marrow organoids [75], all of which are capable of simulating bone morphology, structural organisation and mineralisation processes [96]. Ouyang et al. successfully generated osteo‐callus organoids using MSCs, employing digital light processing (DLP) 3D printing technology combined with stepwise induction methods [87]. Hofmann et al. utilised BMSCs and scaffold‐free 3D self‐organisation techniques to construct woven bone organoids, providing a robust in vitro platform for studying early bone formation [97]. Klein's team successfully developed bone marrow organoids, replicating the structural organisation of bone marrow, particularly the vascular‐like network and supporting haematopoietic cell development [98]. Currently, cutting‐edge research on bone organoids focuses on the application of novel biomaterials and advancements in biofabrication technologies. The objective is to accurately mimic the macroscopic and microscopic architecture of bone tissue—ensuring good mechanical performance at the macroscopic level while integrating a fully functional bone marrow microenvironment at the microscopic level. Su et al. designed a new bioink for bone tissue engineering, composed of gelatin methacrylate (GelMA), alginate methacrylate (AlgMA) and hydroxyapatite (HAP). By co‐culturing this bioink with BMSCs and utilising DLP‐based bioprinting technology, they successfully constructed centimetre‐scale functional bone organoids with excellent mechanical properties. This model precisely replicates the complex microscopic structure of bone tissue, while enabling BMSCs to differentiate into various bone marrow cell types, including haematopoietic cells, immune cells, vascular endothelial cells and chondrocytes, thereby achieving a faithful simulation of the bone marrow microenvironment [99]. Additionally, Zhu et al. developed a dynamic DNA/GelMA hydrogel for constructing woven bone organoids, successfully recapitulating the key biochemical and mechanical characteristics of the bone ECM [100].

The applications of bone organoids can be categorised into four subtypes: physiological, pathological, structural and interactive bone organoids. Physiological bone organoids provide an effective 3D in vitro platform for studying the mechanisms of normal bone differentiation and development. Pathological bone organoids serve as models for simulating disease progression, making them valuable for disease mechanism research, high‐throughput drug screening and personalised treatment strategies. Structural bone organoids, owing to their excellent structural and functional biomimicry, are applicable in bone mechanical performance assessment, tissue engineering and injury repair. Interactive bone organoids represent an advanced and emerging form of bone organoid research, still in its infancy. The goal is to establish structural and functional interactions between bone organoids and other organ systems, such as neural tissue, vascular networks and the endocrine system. Moving beyond the traditional isolated study of bone organoids, this approach aims to reconstruct an integrative multi‐system organoid model that more accurately reflects the human body, facilitating systemic and regenerative medicine research. Current research predominantly focuses on pathological bone organoids, particularly the role of subchondral bone alterations in OA pathogenesis and progression, which remain poorly understood. Elucidating the mechanisms of subchondral bone degeneration in OA is crucial for developing novel therapeutic approaches. Existing studies indicate that excessive activation of osteoclasts in subchondral bone promotes the formation of H‐type blood vessels and alters the joint oxygen microenvironment, which are key drivers of OA progression. Notably, maintaining a hypoxic microenvironment in subchondral bone has been shown to effectively slow down cartilage degeneration in OA [101].

### 3.3 Synovial Organoids

In arthrosis organoids, the synovial membrane secretes synovial fluid, which surrounds various parts of the joint. Synovial fluid is an essential component of the ECM within arthrosis organoids. The synovial membrane consists of multiple cell types, with fibroblasts and macrophages playing key roles in regulating synovial fluid secretion and immune responses. Fibroblasts secrete ECM components and various cytokines, while macrophages exhibit phagocytic activity, removing metabolic waste from the joint cavity [102]. The successful construction of synovial organoids depends on incorporating these two functional cell types. Therefore, synovial organoid construction requires the co‐culture of SFs, vascular endothelial cells and immune cells (e.g., macrophages) to mimic the multicellular interactions of the synovial tissue. Current research predominantly utilises primary synovial cells or synovial cells differentiated from iPSCs as seed cells [103]. The construction of synovial organoids also relies on in vitro culture techniques, including microfluidic technology, 3D culture and 3D bioprinting to simulate interactions between synovial cells and their surrounding microenvironment, ultimately forming organoids that resemble synovial tissue. Sun et al. developed a gene‐edited synovial organoid model, demonstrating that regulating the ECM metabolic network can significantly inhibit joint degeneration [104]. Meanwhile, Martin et al. created the first vascularised synovial cell‐based organ‐on‐a‐chip model, integrating 3D microfluidic technology to simulate the synovial microenvironment. They also applied mechanical loading to mimic joint movement stress, successfully replicating synovial fluid secretion and inflammatory response features [105].

Pathological synovial organoids represent the primary research focus. In rheumatoid arthritis (RA), synovial cells exhibit excessive proliferation and secrete large quantities of inflammatory factors, contributing to joint cartilage and bone destruction. As a result, synovial organoids are widely used to study the pathological mechanisms of arthritis, particularly in conditions like RA [106]. Using synovial organoid models, researchers can observe functional changes in synovial cells under various conditions, including high glucose environments, hypoxia, gene editing or immune stimulation, and explore their specific effects on the joint microenvironment [107]. Chen et al. demonstrated that Sesamol, functioning as a p53 stabiliser, can alleviate RA progression and inhibit synovial organoid growth [108]. Beyond disease mechanism research, synovial organoids also play a key role in therapeutic development. They have been used to evaluate anti‐inflammatory drugs, such as JAK inhibitors and biological agents targeting TNF‐α, demonstrating superior predictive capability compared with traditional 2D cell models [105].

### 3.4 Skeletal Muscle Organoids

Skeletal muscle is a critical functional component of joints, attaching to bone via tendons and serving as the primary executor of movement. By converting chemical energy into mechanical work, it indirectly participates in joint motion, and its force generation and range of activity directly affect joint mobility and stability. Consequently, skeletal muscle organoids are an essential part of joint organoid models. Based on human hPSCs, specific induction and differentiation strategies can yield myogenic progenitor cells and satellite cells (SCs), which then mature into functional skeletal muscle organoids. Mavrommatis et al. employed microfluidic chip technology and stepwise induction to differentiate hPSCs into mature skeletal muscle organoids [109]. Similarly, Jo et al. used a 3D culture approach to induce hPSCs into mature skeletal muscle organoids, demonstrating an arrangement and formation of muscle fibre structures reminiscent of in vivo skeletal muscle [110]. Bioreactors and other 3D dynamic culture systems have been successfully applied in constructing skeletal muscle organoids because they provide mechanical stress stimulation that more accurately simulates the in vivo state of skeletal muscle, thereby enhancing organoid contractility and stability. For instance, Tiburcy et al. utilised hPSCs to build skeletal muscle organoids and employed a bioreactor to apply mechanical loading and evaluate contractility throughout the organoid formation process. Physiological skeletal muscle organoids can be applied to research on muscle repair and regeneration. SCs are crucial in the repair of muscle injuries, and skeletal muscle organoids offer an excellent in vitro platform for investigating SCs proliferation, differentiation and apoptosis [111]. Structural skeletal muscle organoids are primarily used to assess the mechanical properties of muscle; by applying physical, chemical or biological stimuli, researchers can measure contractile force, response time and endurance [112]. Such stimuli—administered topically or via injection—are also significant for drug and therapeutic screening. Meanwhile, pathological skeletal muscle organoids can be employed in the study of degenerative muscle diseases, such as Duchenne muscular dystrophy, amyotrophic lateral sclerosis and mitochondrial myopathies. By using in vitro gene editing methods (e.g., CRISPR‐Cas9) on stem cells, one can construct pathological skeletal muscle organoids for disease modelling, followed by drug screening and therapeutic development [113]. In another example, Raven et al. introduced the recombinant human growth hormone gene into murine myoblasts and, via targeted induction, developed skeletal muscle organoids that serve as a platform for screening gene therapy strategies for muscle diseases [114].

### 3.5 Ligament Organoids

Ligaments are dense fibrous connective tissue bundles that span between two bones, enhancing joint stability and restricting excessive movement. When external force is applied, ligaments help maintain joint alignment, preventing abnormal bone displacement and safeguarding the joint from injury. Consequently, ligament organoids constitute a critical component for preserving the structural and functional integrity of joint organoids [115]. Tzeng et al. successfully constructed periodontal ligament organoids via a stepwise induction strategy [116]. In joints, tendons share similar composition, structure and function with ligaments; therefore, methodologies for constructing tendon organoids can offer valuable guidance for ligament organoid development. For instance, Docheva et al. induced tendon stem/progenitor cells from Achilles tendon biopsy samples to form 3D tendon organoids in vitro, subsequently using these organoids to investigate the mechanisms underlying tendon aging and degeneration [117]. Pathological ligament organoids hold broad potential in regenerative medicine, particularly in the study of sports injury repair. In vitro constructed ligament organoids can accurately model the pathological states of ligament injuries, providing a platform for the development of novel repair materials and therapeutic strategies. In addition, for ligament injuries induced by chronic inflammation or immune factors, ligament organoids facilitate the exploration of disease mechanisms and potential therapeutic targets [118].

## 4 Construction of Osteoarthritis Organoids

Existing OA models include animal models, in vitro cell models, in vitro tissue models and organ‐on‐a‐chip models, but these models fail to simulate the multifactorial interactions of OA and cannot accurately replicate human pathological characteristics, highlighting the urgent need for new OA models [119]. OA organoids represent a multifunctional, multi‐purpose and intelligent artificial organ model that, when constructed in vitro, incorporates cartilage, synovium and subchondral bone into a 3D microstructure while applying pathogenic factors to simulate the pathological process of OA (Table 1).

**TABLE 1:** Comparative analysis of osteoarthritis modelling systems: Traditional preclinical platforms vs. OA organoids.

Comparison dimension	Animal models	In vitro cell models	In vitro tissue models	Organ‐on‐chip models	OA organoids
Core Functionality	Whole‐joint simulation	Molecular mechanism studies	Native tissue architecture	Multi‐tissue integration	3D multi‐tissue pathological simulation
Pathological Fidelity	Moderate (Species divergence > 30%)	Low (Monolayer culture)	High (ECM preservation)	Medium (Single‐pathogen simulation)	High (Human genetic/epigenetic accuracy)
Dynamic Monitoring	Long‐term observation	Static detection	Short‐term testing	Mid‐term tracking (1–4 weeks)	Full‐process tracing (Molecular/cellular resolution)
Microenvironment	Native physiological context	2D artificial matrix	Avascular static system	Microfluidic environment	Smart‐responsive dynamic niche
Technical Complexity	Moderate (surgical modelling)	Low (standard protocols)	High (viability maintenance)	Extreme (microfabrication)	High (Multi‐lineage coculture)
Cost Efficiency	High	Low	Medium	Extreme	Medium‐high (Automated systems)
Drug Screening Utility	In vivo validation (Phase II–III)	Primary screening (Phase 0–I)	Permeability/toxicity tests	Single‐target studies	Preclinical precision screening (Phase I–II)
Key Limitations	Species divergence, Ethical constraints	Microenvironment deficiency, No dynamic crosstalk	Short viability, Static pathology	Long‐term instability, Tissue coordination challenges	Standardisation hurdles

**FIGURE 4:**
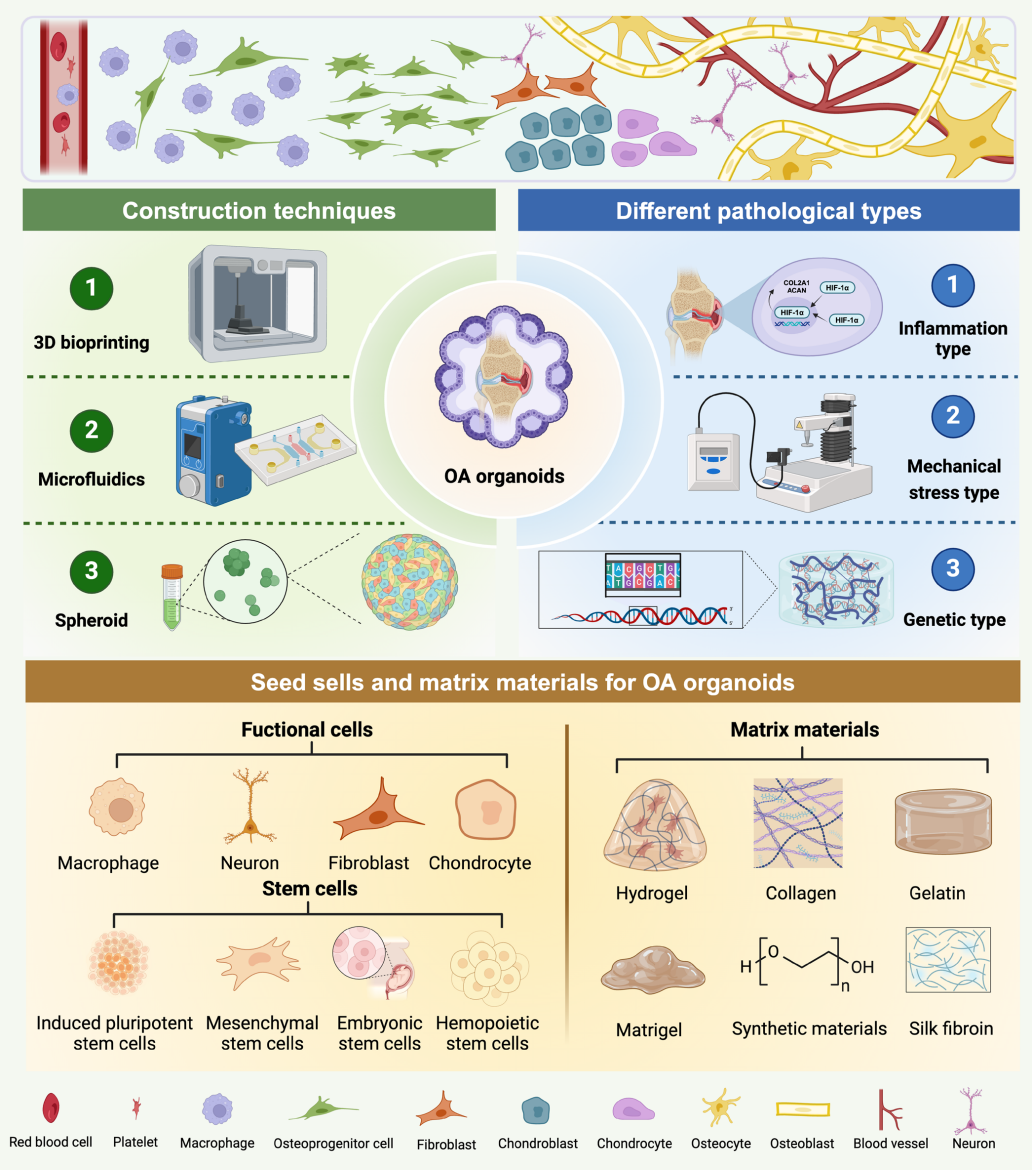
Construction strategies and key technologies for osteoarthritis organoids. Illustrating different seed cell sources, matrix materials, pathological subtypes and core methods such as 3D printing, microfluidics and spheroid self‐assembly. Created with BioRender.com.

As an essential component of organoid research, OA organoids studies are still in their early stages of development. This section will systematically elaborate on the construction strategies of OA organoids from four key aspects: seed cells, matrix materials, pathological types of osteoarthritis organoids and construction techniques (Figure 4).

### 4.1 Seed Cells for Osteoarthritis Organoids

To construct organoids that authentically reflect OA pathological characteristics, it is essential to use stem cell regulation strategies tailored to OA progression and construction strategies specific to pathological organoids. Through directed cell induction and tissue engineering technologies, OA microenvironments can be accurately reconstructed, ensuring the morphological, functional and pathological reliability and stability of OA organoids. Due to their multi‐lineage differentiation potential and self‐renewal capacity, stem cells serve as the core cellular source for the construction of OA organoids [120]. Based on differentiation stage and origin, they are primarily classified into pluripotent stem cells (PSCs) and MSCs [121, 122, 123].

#### 4.1.1 Pluripotent Stem Cells

PSCs encompassing ESCs and iPSCs [124], can precisely recapitulate bone and cartilage development from the ectoderm stage onward through directed differentiation strategies. Among these, ESCs—established as the earliest pluripotent cell line—display strong developmental fidelity and notable in vitro stability and scalability, making them an ideal tool for organoid construction [125, 126]. ESCs are capable of differentiating into multiple types of organoids, including those of the brain [127], liver [128], heart [129] and kidney [121]. However, obtaining ESCs is relatively challenging because it requires the use of early‐stage embryos or trophoblastic cells. The involvement of early embryos raises significant ethical concerns, which limit the application of ESCs in organoid construction [130]. iPSCs represent a groundbreaking advancement in tissue engineering, as they enable the reprogramming of adult cells into a pluripotent state through transcription factor induction, granting them the ability to differentiate into various cell types [131]. iPSCs can differentiate into organ‐specific organoids, mimicking the complex structure and function of native organs. They also offer advantages such as a broad donor availability, lack of ethical concerns and stable genetic integrity. The advent of iPSCs, achieved through somatic cell reprogramming via defined transcription factors, has revolutionised the field by maintaining equivalent pluripotency while circumventing the embryo‐derived ethical controversies inherent to ESCs utilisation. However, it is important to acknowledge that while iPSCs eliminate the need for embryonic materials, ethical considerations persist regarding their biospecimen sourcing protocols and translational applications. These include ensuring informed consent for somatic cell donations, addressing potential commercialisation challenges of personalised cell lines and establishing regulatory frameworks for clinical‐grade iPSCs derivation. This evolving ethical landscape necessitates continued discussions to balance scientific progress with responsible innovation as iPSC‐based organoid technologies advance toward therapeutic implementations [132, 133].

iPSC‐based organoid research has made significant strides in disease modelling, drug development and regenerative medicine. Organoids derived from iPSCs have been successfully developed for vascular [134], pulmonary [135], cardiac [129] and intestinal tissues [136], among others. iPSCs also serve as an optimal cellular source for arthrosis organoid construction. Under specific growth factors and 3D culture systems, iPSCs can efficiently differentiate into chondrocytes, producing an extracellular matrix rich in glycosaminoglycans (GAGs) and type II collagen. Additionally, iPSCs can be directed into osteogenic differentiation through mesodermal induction protocols, upregulating bone marker genes such as COL1A1 and BGLAP and forming mineralised matrices [137]. Shannon et al. successfully developed bone and cartilage organoids from mouse‐derived iPSCs, replicating endochondral ossification and osteogenesis [138]. Gabriella et al. demonstrated that iPSC‐derived chondrocytes could form cartilage‐like nodules under scaffold and scaffold‐free conditions, integrating with host cartilage to facilitate defect repair, thereby validating the self‐organising properties of cartilage organoid formation [139]. Khan et al. induced iPSCs to differentiate into mesenchymal, endothelial and haematopoietic lineage cells, successfully replicating the complex microenvironment of human bone marrow [140]. OA organoids consist of a cartilage–subchondral bone–synovium composite structure, requiring differentiation‐specific regulatory strategies for seed cells to guide stem cell‐directed differentiation into cartilage, subchondral bone and synovial layers. Cartilage layer induction can be achieved using iPSCs combined with TGF‐β3 and BMP‐2 to establish a cartilage‐specific differentiation system, activating the SOX9/COL2A1 pathway for chondrogenic regulation [141, 142]. Subchondral bone layer induction can be performed by dual‐signal activation of the BMP‐2/BMP‐7 osteogenic differentiation pathway, coupled with mechanical loading to promote mineralisation [143, 144].

#### 4.1.2 Mesenchymal Stem Cells

MSCs, with their multipotent differentiation potential and ease of isolation, are highly valuable for constructing bone and cartilage organoids [145]. MSCs are widely found in bone marrow, adipose tissue, umbilical cord, placenta and dental pulp. Under specific induction conditions (e.g., TGF‐β, BMP‐6), they can differentiate into osteoblasts or chondrocytes and secrete key matrix components such as osteocalcin and type II collagen [146, 147, 148]. In bone organoid construction, BMSCs are often combined with bioactive materials (e.g., bone matrix‐inspired bioinks, calcium phosphate ceramic scaffolds) to enhance bone formation and vascularisation through self‐mineralisation or co‐delivery of endothelial cells/growth factors [149]. Su et al. successfully constructed self‐mineralising bone organoids using BMSCs and bone matrix‐induced bioinks, which exhibited natural bone‐like structures after transplantation in nude mice [99]. Martin et al. recreated the bone marrow microenvironment using BMSCs and human haematopoietic stem cells (HSCs) [150]. Additionally, periosteum‐derived stem cells are preferred for bone repair due to their sensitivity to mechanical stimuli, while adipose‐derived stem cells are gaining attention for their abundance, low immunogenicity and stable proliferation [151]. Cartilage organoid construction relies on high‐density culture and microenvironment simulation techniques. For example, GelMA microspheres or 3D scaffolds combined with TGF‐β3 induction can promote MSC differentiation into functional chondrocytes and the formation of layered cartilage structures. Although BMSCs remain the primary choice, adipose‐derived MSCs show comparable chondrogenic differentiation efficiency and are easier to obtain [90]. Notably, the immunomodulatory function of MSCs can improve the local microenvironment by inhibiting inflammatory factors (e.g., IL‐1β in osteoarthritis), indirectly promoting organoid maturation [148, 152]. However, differentiation efficiency and stability vary among MSC sources. For example, dental pulp stem cells excel in vascularised dental pulp organoid construction but require optimised pre‐differentiation strategies to avoid proliferation inhibition [149, 153].

#### 4.1.3 Haematopoietic Stem Cells and Their Progeny

HSCs are a unique population within the bone marrow, possessing both self‐renewal and multipotent differentiation capacities that enable the continual regeneration of all lineages in the adult blood and immune systems [154, 155]. They retain this distinctive self‐renewal ability throughout the lifespan, making them the key cell type responsible for maintaining haematopoietic homeostasis [156]. Among the progeny of HSCs are various lineage progenitor cells—including myeloid progenitor cells (MkPs), lymphoid progenitor cells (EpPs) and endothelial progenitor cells (EPCs)—which under specific conditions can further differentiate into mature blood cells such as erythrocytes, leukocytes, platelets and vascular endothelial cells [157, 158]. By culturing HSCs and their progeny in vitro, researchers can construct blood organoids that recapitulate human haematopoiesis for disease modelling and drug screening. Additionally, these cells can be used to build immune organoids, thereby facilitating the study of immune system development and function [159]. EPCs are a pluripotent cell population capable of differentiating into mature ECs, playing a crucial role in vascular regeneration and repair. EPCs can be classified into early EPCs and late EPCs: Early EPCs exhibit higher proliferative capacity, whereas late EPCs display more mature endothelial characteristics [160]. Biphasic differentiation of EPCs refers to their ability to differentiate into synovial fibroblasts or endothelial cells, regulated by multiple signalling pathways [161]. Differentiation into synovial fibroblasts is associated with Notch signalling activation, which has been shown to promote EPC‐to‐synovial cell differentiation in both in vivo and in vitro studies [162]. In contrast, differentiation into endothelial cells is primarily driven by VEGF and SDF‐1, which enhance EPCs migration and proliferation, leading to the formation of a mature endothelial network [161]. By precisely controlling the synovial–vascular lineage biphasic differentiation of EPCs, it is possible to generate mature synovial fibroblasts and endothelial networks, facilitating the construction of the synovial layer in OA organoids. Synovial layer construction can be facilitated through directed induction of EPCs by TGF‐β1 and PDGF‐BB, generating synovial fibroblasts and vascular endothelial cells, while incorporating macrophages to simulate the inflammatory microenvironment [163].

#### 4.1.4 Functional Cells

In the preparation of OA organoids, functional cell populations beyond stem cells are also pivotal contributors by imparting distinct pathological environments. Derived from MSCs, osteoblasts synthesise and mineralise the bone matrix, secreting key proteins such as collagen and osteocalcin [164]. In organoid construction, osteoblasts can be obtained by inducing the MC3T3‐E1 cell line differentiation and participate in trabecular bone formation [165]. Originating from HSCs (e.g., monocyte/macrophage lineage), osteoclasts differentiate via the RANKL‐RANK signalling pathway [164]. Osteoblast‐secreted RANKL and macrophage colony‐stimulating factor are critical for osteoclast differentiation [166]. The interplay between osteoblasts and osteoclasts is essential for bone repair, regeneration and the study of osteoporosis and osteoarthritis [167]. Metcalfe et al. constructed micrometre‐scale bone organoids where osteoclast overactivation simulated pathological bone loss (e.g., osteoporosis) and co‐culture systems with osteoblasts modelled bone remodelling imbalances [96]. Comprising 90%–95% of adult bone cells, osteocytes are embedded in the mineralised matrix and regulate osteoblast/osteoclast activity through dendritic networks, acting as mechanical stress sensors [166, 168]. Incorporating osteocytes enhances organoid physiological relevance by simulating bone microenvironment signalling (e.g., RANKL, OPG) [169]. Chondrocytes maintain cartilage structure by secreting type II collagen and aggrecan and differentiate into osteoblasts during endochondral ossification [165, 170]. Co‐culturing chondrocytes with endothelial cells can simulate the articular cartilage–bone interface, useful for studying cartilage degeneration and ectopic ossification in osteoarthritis [171]. Simulating pain perception additionally relies on the incorporation of neurons within the OA organoids [172]. Notably, macrophages and other immune cells function as key contributors within OA organoids, facilitating essential immune responses and shaping disease‐related processes. M1 macrophages are the primary pro‐inflammatory cells in synovitis, activated through multiple signalling pathways (e.g., NF‐κB, MAPK) and secreting large amounts of pro‐inflammatory cytokines, including IL‐1β, TNF‐α, IL‐6, IL‐12, CCL2 and CCL5 [173]. These cytokines not only sustain the inflammatory response but also exacerbate inflammation through interactions with T cells, neutrophils and monocytes [174]. Additionally, M1 macrophages release reactive oxygen species and nitric oxide, directly damaging joint tissues. They also interact with synovial fibroblasts, further promoting inflammatory cytokine secretion and overexpression of inflammatory mediators [175]. In synovial inflammation, the polarisation of M1 macrophages and the abnormal activation of synovial fibroblasts play key roles in cytokine cascade release, MMP overexpression and inflammatory mediator upregulation. Incorporating macrophages and synovial cells in OA organoids enhances the simulation of the OA inflammatory microenvironment.

### 4.2 Matrix Materials for Osteoarthritis Organoids

In the construction of OA organoids, matrix materials are crucial in supporting cellular components, providing a 3D growth framework to mimic the natural bone development environment. These materials supply sufficient nutrients essential for maintaining stemness and stable differentiation pathways, while also promoting cell growth, proliferation, differentiation, migration and interaction [176]. Additionally, matrix materials help ensure mechanical strength, structural integrity and elasticity of the organoid. Among these materials, hydrogels—composed of a three‐dimensional polymer network with high water content (typically over 70% of total weight)—offer excellent biocompatibility, tunable mechanical properties and bioactivity, making them particularly significant in OA organoid construction [177, 178]. The application of matrix materials varies depending on the specific tissue components within OA organoids. The following sections will introduce the matrix materials used in constructing articular cartilage, subchondral bone and synovial tissue.

#### 4.2.1 Matrix Materials for Cartilage Tissue Engineering

The matrix materials required for cartilage tissue engineering include various natural and synthetic hydrogels such as collagen, gelatin and hyaluronic acid (HA), as well as polyethylene glycol (PEG), poly(lactic‐*co*‐glycolic acid) (PLGA) and bioactive molecules. Through different processing methods and combinations, these materials can form highly biocompatible and bioactive scaffolds, providing a diverse selection for cartilage tissue engineering. Gelatin, a partial hydrolysis product of collagen, retains the arginine‐glycine‐aspartic acid (RGD) sequence. It provides dynamic mechanical stimulation in hydrogel form, promoting cell migration and matrix deposition and exhibits excellent biocompatibility and ECM similarity, making it widely used in cartilage organoids construction [179]. Yang et al. developed gelatin‐based micro‐cryogels that achieved synchronous regeneration of cartilage and bone tissues in organoids, with degradation rates matching tissue regeneration speeds [180]. Additionally, hydrogels are widely used in cartilage tissue engineering [181, 182]. Su's team highlighted that silk fibroin‐based hydrogels not only possess an ECM‐like structure but also exhibit unique mechanical properties and excellent biocompatibility, making them an ideal biomaterial for cartilage tissue construction [183]. Microspheres prepared from hydrogels offer tunable physical, chemical and biological properties, effectively mimicking the 3D ECM‐like scaffold required for cartilage growth. In particular, Su et al. developed RGD‐silk fibroin‐DNA hydrogel microspheres (RSD‐MS), which demonstrated favourable material properties and significantly enhanced cartilage regeneration in in vivo studies [92]. Hydrogels can also serve as carriers for multiple bioactive factors and exosomes, enabling them to simulate various physiological and pathological environments. Zhang et al. utilised 3D bioprinting hydrogel combined with exosomes loaded with chondrogenic stimuli agents, which exhibited notable immunomodulatory effects. This faster and safer biomaterial promotes cartilage proliferation and repair, making it highly applicable in the treatment of osteoarthritis and other cartilage‐related diseases [184]. PLGA is a biodegradable copolymer composed of lactic acid and glycolic acid. With excellent biocompatibility and mechanical strength, PLGA provides a robust structural environment conducive to organoid development. Its degradation rate can be fine‐tuned by adjusting the lactic acid‐to‐glycolic acid ratio, ensuring synchronisation with cell growth and differentiation. Chen et al. developed PLGA matrix loaded with a cell‐free adipose extract, which effectively facilitated the formation of cartilage organoids and promoted chondrocyte differentiation [185].

#### 4.2.2 Matrix Materials for Subchondral Bone Tissue Engineering

Appropriate matrix materials play a crucial role in actively inducing subchondral bone mineralisation and osteogenesis, providing essential support for bone tissue formation and function. These materials are indispensable for developing functional subchondral bone tissue. A wide variety of matrix materials are used for subchondral bone construction, which are similar to the matrix materials for cartilage tissue engineering, including collagen, Matrigel, hydrogels, alginate, hydroxyapatite, chitosan, HA, bioactive glass, polylactic acid and microporous materials. Collagen is the primary organic component of the bone ECM, with type I collagen comprising approximately 90% of the total bone matrix. It is frequently used as a scaffold base to mimic the 3D bone microenvironment and promote MSCs differentiation into osteoblasts. Matrigel, derived from the basement membrane of Engelbreth‐Holm‐Swarm mouse sarcoma, primarily consists of laminin, collagen IV, heparan sulphate proteoglycan (perlecan) and various growth factors such as fibroblast growth factor, epidermal growth factor and transforming growth factor‐β [186]. In a study by Enyi et al., Matrigel was used to support BMP9‐induced osteogenic differentiation and bone matrix mineralisation, demonstrating its significance in alveolar bone cell differentiation and bone tissue engineering [187]. These components form a three‐dimensional network structure similar to natural ECM, providing an excellent environment for cell attachment and growth, and supporting 3D culture and osteogenic/chondrogenic differentiation of stem cells [188]. Hydrogels, due to their excellent biocompatibility and tunable mechanical properties, have emerged as key materials in bone tissue engineering, serving as scaffolds to support cell growth and differentiation. GelMA hydrogels, known for their adjustability and biocompatibility, have been extensively studied for musculoskeletal tissue regeneration [189]. For instance, self‐assembling peptide hydrogels exhibit exceptional performance in bone regeneration, facilitating cell adhesion, migration, proliferation and differentiation [190]. Su et al. developed an ECM‐DNA‐CPO‐based engineered biomimetic hydrogel with a dynamic network to simulate the bone microenvironment, facilitating the construction of vascularised and mineralised bone organoids [191]. The 3D printing technology has played a significant role in the fabrication and application of hydrogel‐based materials. By leveraging 3D printing, researchers can integrate extracellular matrix components within hydrogels, creating adequate spatial structures for osteogenesis and chondrogenesis, thereby promoting tissue repair [192]. Additionally, DNA hydrogels, recognised for their programmability, tunability and mechanical properties, are considered a promising innovation in bone tissue engineering. Su's team highlighted the potential of DNA‐functionalised bio‐ink combined with 3D bioprinting to construct bone tissue organoids, showcasing its prospects in bone regeneration and osteoarthritis treatment [193]. PEG is a highly biocompatible, water‐soluble polymer characterised by exceptional hydrophilicity and biological inertness, making it widely applicable in tissue engineering and organoid construction [194]. Chemical modifications, such as the introduction of reactive functional groups, enhance PEG's interactions with cells and allow for mechanical property adjustments, thereby improving its practical utility in tissue engineering. Ehrbar et al. developed a hybrid hydrogel by combining PEG with HA and utilising a transglutaminase crosslinking mechanism. This significantly improved the physical and biochemical properties of the hydrogel, providing an optimal microenvironment for the development of humanised bone marrow organoids [150].

#### 4.2.3 Matrix Materials for Synovial Tissue Engineering

The key to constructing synovial tissue lies in accurately mimicking its natural microenvironment, where the selection of matrix materials—particularly hydrogels—directly influences cellular behaviours such as proliferation, differentiation and functional expression. The choice of synovial tissue matrix materials must meet biomechanical requirements, enabling adaptation to mechanical compression, loading and movement‐related forces acting on synovial tissue. By designing scaffold materials with specific mechanical properties, researchers can promote synovial cell growth and differentiation while effectively simulating the natural mechanical environment of synovial tissue [195]. Natural hydrogel materials include collagen, fibrin and hyaluronic acid, while synthetic materials such as polylactic acid and PLGA, along with composite materials like chitosan/hydroxyapatite, are also commonly used. As the core matrix material for synovial tissue, hydrogels must balance biomimicry, tunability and functionality. Guo et al. utilised synovial MSCs as seed cells and employed a chitosan hydrogel/3D‐printed poly(ε‐caprolactone) hybrid scaffold, combined with tetrahedral framework nucleic acid recruitment, to successfully regenerate both cartilage and synovial tissue [196]. Dai et al. developed a senescence‐targeted miR‐24 μS/SMSC organoid hydrogel for synovial organoid construction. This material demonstrated excellent chondrogenic potential in vitro, while animal experiments showed superior cartilage repair at 24 weeks, better joint function maintenance and reduced intra‐articular inflammatory response following rat joint transplantation [197]. Peter et al. successfully constructed a synovial organoid‐on‐a‐chip using a composite matrix composed of Matrigel, PEG–dextran and fibrin hydrogels. This platform enables monitoring of tissue‐level remodelling under arthritic pathological conditions [198]. Future research will focus on dynamically responsive materials, multi‐scale structural design and clinical translation, providing a more reliable material foundation for precise synovial tissue construction.

### 4.3 Pathological Types of Osteoarthritis Organoids

OA involves multiple factors, including mechanical stress, inflammation and genetic susceptibility. The lack of an ideal research model is a key bottleneck restricting OA mechanism research, drug development and personalised treatment. Wei et al. constructed an OA organoid model using PSCs carrying dual fluorescent markers COL2A1‐mCherry/COL10A1‐eGFP. Through high‐throughput screening of 2040 FDA‐approved drugs, they identified that the α‐adrenergic receptor (α‐AR) antagonist phentolamine promotes chondrogenesis, inhibits hypertrophy and protects cartilage organoids from degeneration [199]. In the field of personalised medicine, Arrigoni et al. developed an OA organoid‐on‐a‐chip model by isolating chondrocytes and synovial fibroblasts from OA patients and inducing an OA microenvironment through the addition of OA synovial fluid, which stimulated inflammatory cytokine and degradative enzyme production. By using patient‐matched cells and synovial fluid, this personalised OA organoid model was applied to screen orthopaedic biologics for patient‐specific efficacy [200]. The construction of OA organoids requires differentiated models tailored to distinct pathogenic mechanisms. Based on the three primary causes of OA, OA organoid models can be constructed from three subtypes: inflammatory subtype, mechanical stress‐induced subtype and genetic predisposition subtype.

#### 4.3.1 Inflammatory Type of Osteoarthritis Organoids

The inflammatory OA organoid model is primarily driven by immune system dysregulation and chronic inflammation, characterised by immune cell infiltration and a pro‐inflammatory cytokine cascade. The core mechanisms involve T‐cell, B‐cell and macrophage‐mediated synovial inflammation, leading to abnormal secretion of pro‐inflammatory factors such as IL‐1β and TNF‐α, which in turn trigger cartilage matrix degradation (upregulation of MMP‐13 and COX‐2), joint effusion and accelerated chondrocyte apoptosis, ultimately resulting in cartilage destruction and joint stiffness [201]. Using iPSCs co‐cultured with a matrix gel embedded with pro‐inflammatory factors makes it possible to emulate an inflammatory microenvironment. Currently, Matrigel loaded with inflammatory factors—such as programmable DNA hydrogels, nanocomposite hydrogels and smart responsive hydrogels—has attracted significant research attention [89]. Notably, programmable DNA hydrogels exhibit unique programmability compared with other natural and synthetic polymer hydrogels, allowing precise structural customisation and tunable properties [202]. Targeted DNA segments can be generated via hybridisation chain reactions, PCR amplification or other methods and subsequently assembled stepwise into an ordered DNA‐based matrix under electrostatic interactions. Through careful sequence design, researchers can tailor the matrix's physicochemical properties—such as rigidity, distribution of active sites, topological texture and creep behaviour—thus achieving fine control over the hydrogel's characteristics [203]. By combining programmable DNA hydrogels with other matrix materials, it is possible to effectively mimic cartilage, subchondral bone and synovial matrices to regulate the inflammatory differentiation of stem cells. Specifically, inducing inflammation in stages typically entails a multi‐step cascade. First, a low concentration of pro‐inflammatory factors is applied to initiate a baseline inflammatory response. Subsequently, additional factors are introduced stepwise to replicate progressively more complex inflammatory environments. Treating cells with low‐concentration IL‐1β for 24 h induces a baseline inflammatory response. Subsequently adding TNF‐α and IL‐6 simulates a progressively complex inflammatory microenvironment. Keishi et al. developed an inflammatory microenvironment model for RA fibroblast‐like synoviocytes by using a stepwise cytokine induction approach, where cells were sequentially stimulated with TNF‐α, IL‐1β and IL‐6, effectively recapitulating the inflammatory cascade of RA in vitro [204].

#### 4.3.2 Mechanical Stress Type of Osteoarthritis Organoids

Mechanical stress‐induced OA is primarily caused by excessive or abnormal mechanical loading. Prolonged mechanical overload disrupts the biomechanical balance at the cartilage–bone interface through abnormal stress transmission, potentially leading to microcracks or fractures on the articular cartilage surface, which compromises cartilage structure and load‐bearing capacity, ultimately accelerating cartilage degeneration [205]. Additionally, mechanical pressure contributes to the degradation of cartilage matrix components, such as collagen fibre rupture and proteoglycan loss, while simultaneously inhibiting the repair process, resulting in metabolic imbalance and ultimately leading to joint pain, swelling and functional impairment [206]. The construction of mechanical stress‐induced OA organoids relies on dynamic culture systems, utilising multi‐axial bioreactors to apply cyclic mechanical stress. Load parameters are set, allowing precise control over stress magnitude, frequency and duration. This approach enables researchers to investigate the effects of varying degrees of mechanical stress on osteocytes, chondrocytes and bone organoids, ultimately defining the optimal mechanical environment that induces OA. By systematically adjusting these parameters, researchers can better simulate the physiological and pathological biomechanical environment of joints. A combination of dynamic compression and dynamic shear loading is employed to better replicate the physiological mechanical state of cartilage tissue in vivo. Lisbet et al. conducted studies on high mechanical strain in 3D matrices, aiming to elucidate the biomechanical mechanisms underlying OA progression. Their research analysed gene expression changes in chondrocytes subjected to mechanical dynamic strain stimulation, revealing how mechanical stress influences cellular behaviour [172]. Additionally, electro‐mechanical coupling should be enhanced during culture to augment mechanical stress transmission within OA organoids. This involves the application of electrical stimulation or magnetic field reinforcement, such as low‐intensity pulsed ultrasound or static magnetic fields, to activate Piezo1 ion channels and promote chondrocyte mechanotransduction. These strategies maximise the effects of mechanical stress on OA organoids. Geng et al. demonstrated that LIPUS generates cyclic mechanical waves that improve ischaemia, suppress inflammation and promote cartilage repair. Moreover, LIPUS significantly enhances chondrocyte proliferation and extracellular matrix synthesis while reducing osteoclast activity, further contributing to OA prevention and treatment [207].

#### 4.3.3 Genetics Type of Osteoarthritis Organoids

Genetic predisposition OA involves multifactorial interactions, with gene mutations or polymorphisms playing a pivotal role, alongside factors affecting chondrocyte differentiation, inflammatory responses and extracellular matrix degradation. Specifically, polymorphisms in genes such as ESR1 and GDF5 are closely associated with an increased risk of knee OA, while variations in cytokine‐related genes (e.g., IL‐1β and TGF‐β1) further contribute to OA susceptibility and severity [208]. Additionally, dysregulated immune cell function, cytokine imbalances (e.g., abnormal secretion of TNF‐α and IL‐17) and autoantibody production collectively drive joint inflammation and cartilage destruction [209]. Environmental factors can act as triggers in genetically predisposed individuals, accelerating cartilage degeneration. The core of genetic predisposition organoid models lies in gene editing strategies. The process involves selecting OA‐related target genes, such as RUNX1, HIF‐2α, MAPK12, FOS, MMP‐13 and Sox9, and designing specific single guide RNAs (sgRNAs) based on target gene sequences. The Cas9 protein and sgRNAs sequences are then inserted into expression plasmid vectors, with adeno‐associated viruses (AAV) or lentiviruses serving as delivery systems to introduce the CRISPR/Cas9 system into target stem cells. This enables the knockout of cartilage‐protective genes or overexpression of pathogenic genes. Chen et al. utilised CRISPR/Cas9‐mediated gene knockout and downregulation of MMP‐13, IL‐1β and NGF, significantly mitigating OA‐associated inflammation and cartilage degradation [210]. Kiran et al. replaced TALEN with CRISPR technology, enhancing the efficiency of genome editing in hPSCs and enabling the development of genotype‐specific organoids [211].

### 4.4 Construction Techniques of Osteoarthritis Organoids

The core of OA organoid construction lies in precisely replicating OA pathological features, such as cartilage degeneration, bone remodelling and inflammation, which are typical pathological processes of OA. The extent to which pathological features are replicated will directly impact the reliability of subsequent applications, including disease mechanism analysis and drug screening. The construction of OA organoids involves the integration of multiple engineering methods and biotechnologies, combining cellular components, matrix materials and subtype‐specific induction strategies through engineering approaches. The goal is to fully integrate pathological cartilage organoids, subchondral bone organoids and synovial organoids, ensuring structural and functional coordination among these three components to accurately replicate the pathological characteristics of OA.

#### 4.4.1 3D Bioprinting Technology

3D bioprinting technology represents a revolutionary advancement in tissue engineering, enabling the precise spatial control of cells, biomaterials and bioactive factors to construct complex 3D structures [212]. Bioprinting allows for the fabrication of high‐fidelity arthrosis organoids, with key advantages including high precision and reproducibility in tissue construction [213]. Using high‐precision DLP bioprinting technology, it is possible to layer‐by‐layer print cartilage–subchondral bone–synovium composite structures with exceptionally high spatial positioning accuracy of cells [214]. The stability of organoid structure and function depends on the precise control of multiple factors, including biomaterial preparation, printing speed, temperature and layer thickness [215]. Lode et al. successfully achieved full‐thickness osteochondral tissue regeneration by employing bioprinting technology with bioinks containing primary bone–cartilage substitute cells, in combination with multi‐layered mineralisation constructs [216]. Additionally, 3D bioprinting technology allows for the deposition of bioinks containing BMSCs, ECM and growth factors, integrating multiple biomaterials (e.g., GelMA, AlgMA and hydroxyapatite) to further optimise the structure and function of organoids, achieving mechanical properties highly similar to natural bone tissue. Ouyang et al. developed bone organoids with reparative properties by encapsulating BMSCs in GelMA microspheres using 3D bioprinting, followed by stepwise differentiation induction [87]. Similarly, Su et al. applied 3D bioprinting technology to fabricate self‐mineralising bone organoids using bioinks containing a diverse range of biomaterials [99]. The introduction of nanoparticles provides additional advancements in bioprinting technology, enhancing its capability to construct complex tissue structures. Specifically, nanoparticles improve the precision and functionality of bioprinted tissues, facilitating the accurate replication of the intricate architecture of bone and cartilage [216].

#### 4.4.2 Microfluidic Technology

The application of microfluidic and microporous array chip technology in organoid manufacturing represents a significant advancement in biomedical engineering. By leveraging precisely designed microfluidic chips and modular construction, this technology enables dynamic control of the cell culture environment, effectively mimicking the pathophysiological characteristics of OA while enhancing organoid culture consistency and efficiency. Abbasalizadeh et al. utilised droplet microfluidic technology to continuously generate cell‐laden microcapsules, promoting cell proliferation, self‐contraction and self‐organisation into organoids, ultimately leading to large‐scale development of fully structured liver organoids [217]. Microfluidic and microporous array chips function by creating micro‐scale pores or channels within the chip, allowing for precise simulation of the human tissue microenvironment. By controlling the size and distribution of these pores, researchers can finely regulate intercellular interactions and signal transduction [218], thereby effectively replicating the inflammatory cell interactions and cytokine cascades observed in the pathological microenvironment of OA. Moreover, microfluidic systems can simulate biological processes across multiple organ systems, including vascular, neural, respiratory and digestive systems. For example, microfluidic models have successfully reconstructed the alveolar‐capillary interface, replicating its role in respiration and inflammation [219]. This technology provides crucial support for the construction of cartilage–subchondral bone–synovium interfaces in OA organoid models. Additionally, microfluidic technology facilitates continuous and dynamic organoid culture platforms, enabling researchers to real‐time monitor organoid behaviour and drug responses, thereby optimising drug screening and disease modelling. Khademhosseini et al. proposed a fully integrated modular system incorporating physical, biochemical and optical sensing platforms, which operates organ‐on‐a‐chip units in a continuous, dynamic and automated manner [220]. Regarding biomaterial preparation, microfluidic technology, when combined with other advanced fabrication techniques, enables the development of stem cell culture environments with superior ECM biomimetic properties for OA organoid formation. Su et al. employed photo‐crosslinking and self‐assembly microfluidic integration systems to generate novel RSD‐MS, which served as the foundation for constructing primary cartilage organoids [92].

#### 4.4.3 Spheroid Technology

The application of spheroid technology in organoid construction represents a major advancement in tissue engineering. The core principle lies in utilising the 3D structural properties of spheroids to simulate the microenvironment of human tissues, making it a foundational technology for organoid construction. This is particularly valuable for modelling complex cell–cell and cell–matrix interactions, facilitating advancements in disease modelling, drug screening, tissue engineering and regenerative medicine. By suspending single‐cell layers or multiple cell types in culture medium, cells spontaneously aggregate into 3D spheroids through physical forces such as gravity, surface tension or magnetism. Sequential application of specific cytokines (e.g., FGF, EGF, WNT‐3a, collagen and TGF‐β) gradually induces cell differentiation, mimicking the natural developmental progression from mesoderm to mature cartilage, bone and synovium [221, 222]. Martin et al. cultivated spheroids on low‐adhesion surfaces and subsequently loaded them into an assembly chamber, where acoustic nodal assembly technology was used to generate hepatic spheroids. These spheroids matured in fibrinogen gel, eventually forming larger organ‐like structures [223]. Spheroids recapitulate key aspects of the human tissue microenvironment, including ECM deposition, intercellular interactions and metabolic waste clearance, thereby enhancing the study of cellular behaviour and signal transduction mechanisms [224]. This capability is particularly well‐suited for reproducing the pathological microenvironment of OA. Moreover, spheroid technology integrates closely with 3D bioprinting, where high‐cell‐density bioprinting of spheroids significantly reduces the maturation time required for large tissue/organoid formation [225]. By layered induction of spheroids corresponding to cartilage, subchondral bone and synovium, followed by high‐precision DLP bioprinting, it is possible to assemble pathological cartilage–subchondral bone–synovium composite structures. This integration achieves a structurally and functionally unified OA organoid model, effectively replicating OA pathological features in vitro.

## 5 Intelligent Manufacturing Strategies for Osteoarthritis Organoids

Traditional manual culture methods for organoids rely heavily on clean laboratory environments, are low in efficiency, cost‐intensive and struggle to ensure batch‐to‐batch consistency and stability, limiting their industrial application. Intelligent organoid manufacturing in OA is a closed‐loop cyber‐physical system that integrates biomedical automation (bioreactors, liquid‐handling robots), AI‐driven process control (machine learning‐based optimisation of culture parameters) and multi‐omics feedback (real‐time transcriptomic/metabolomic monitoring). Its OA‐specific features include dynamic joint mechanics simulation, integration of patient‐specific inflammatory biomarkers and AI‐powered cell differentiation monitoring. Through a translational pipeline of ‘clinical data‐customised protocols‐intelligent quality control‐organoid output’, this approach combines computer vision‐based morphological analysis and AI‐driven drug efficacy prediction models to enable ‘human‐free factory’ standardised production, providing a framework for addressing subsequent technological challenges. This approach enables real‐time monitoring and regulation of the culture process, achieving fully automated large‐scale manufacturing with high efficiency and low cost, while maintaining strict quality control for scalable organoid production and clinical translation. Given the disease context of OA, this review define this manufacturing strategy as ‘human‐free factory’ osteoarthritis pathological organoid manufacturing. Addressing the challenges of standardisation and large‐scale culture of pathological organoids, particularly in OA, is crucial for their clinical and translational applications. However, ‘human‐free factory’ manufacturing for OA organoids remains in its early stages, facing multiple technological challenges, including: Limitations of static culture models, which fail to achieve full functional maturity. Insufficient precision of image analysis algorithms for morphological assessment. Difficulties in integrating multi‐omics data, which impedes comprehensive functional evaluation. To address these critical challenges, this review proposes an innovative framework for ‘human‐free factory’ OA pathological organoid manufacturing. The core principle of this strategy is to leverage software‐driven automation to control hardware execution, ensuring a seamless, intelligent organoid production workflow. Our comprehensive discussion of each step in the process provides researchers with a structured roadmap for developing intelligent pathological organoid manufacturing. Moreover, by identifying key technical bottlenecks, this strategy drives multidisciplinary collaboration across fundamental medicine, bioengineering and information technology, fostering technological advancements and cross‐disciplinary research efforts. Ultimately, this approach aims to accelerate the clinical translation and application of ‘human‐free factory’ OA pathological organoid manufacturing, laying the foundation for next‐generation disease modelling, drug discovery and precision medicine.

**FIGURE 5:**
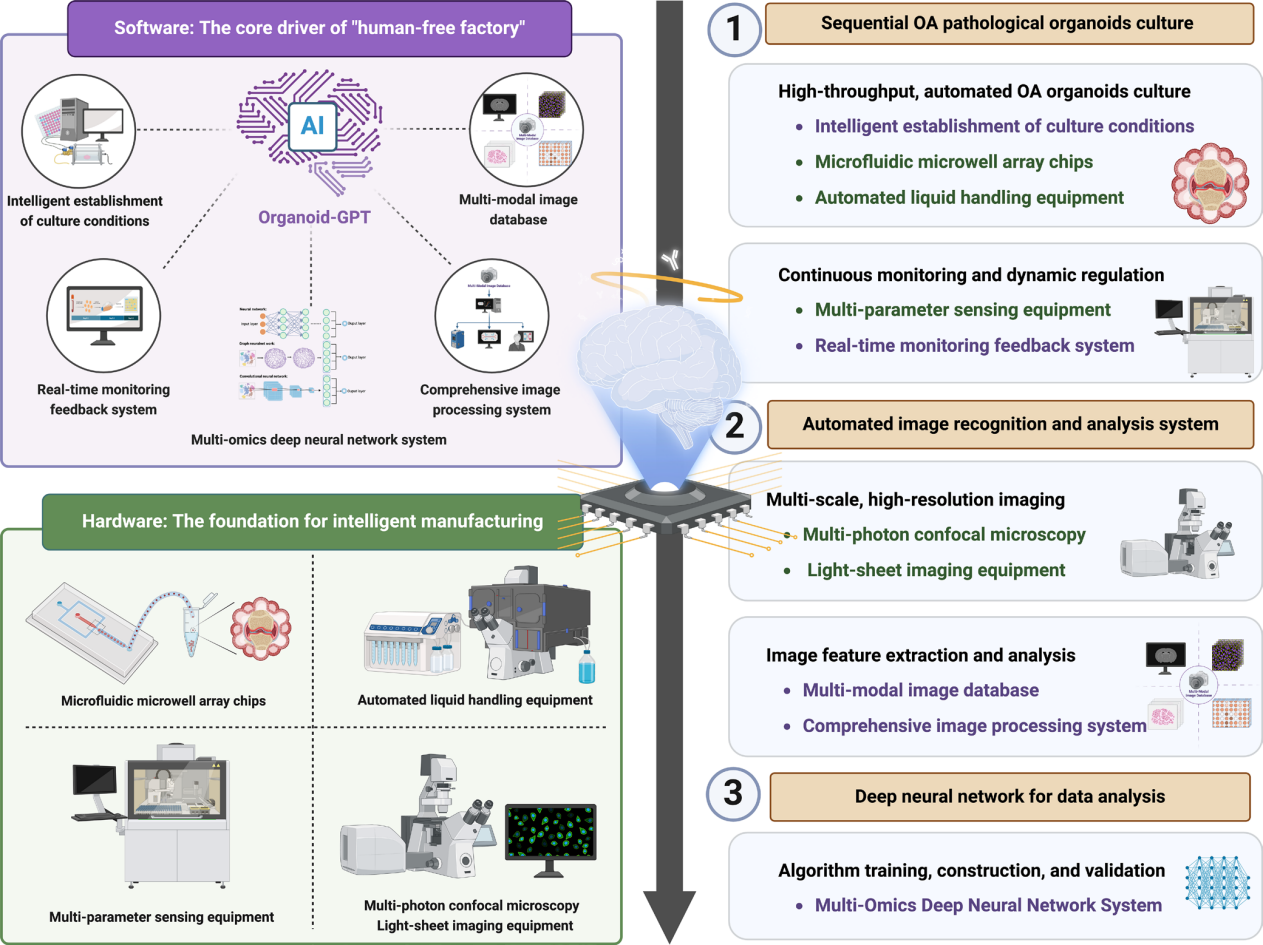
Workflow for the intelligent manufacturing of osteoarthritis organoids. Focusing on an automated culture system, real‐time sensor feedback, image recognition and big‐data analytics, this figure illustrates the ‘human‐free factory’ concept and how it enables a streamlined, intelligent organoid fabrication process. Created with BioRender.com.

Traditional static culture systems fail to accurately replicate the dynamic microenvironment of human joints. The physiological function of osteoarthritic tissues is influenced by multiple factors, including mechanical stimulation, temperature, oxygen partial pressure, pH levels and growth factor gradients. For instance, chondrocytes activate the p38 MAPK signalling pathway under mechanical stress, leading to inflammatory cytokine secretion and nociceptive signalling [172]. However, current culture models lack precise control over these dynamic conditions, resulting in immature OA organoid functionality that fails to fully replicate in vivo joint biology [226]. OA organoids exhibit significant 3D heterogeneity, with complex and highly variable internal structures. However, existing image analysis algorithms struggle to precisely recognise fine microstructural features. While multifocal imaging provides high‐resolution 3D datasets, its slow processing speed and high computational costs hinder its application in high‐throughput organoid morphological assessments [227]. OA organoid research generates vast amounts of multi‐omics data, including genomics, transcriptomics, proteomics and metabolomics. These datasets are often unstructured, semi‐structured or heterogeneous, making data integration and functional evaluation a major challenge [228]. The lack of computational models and AI‐driven analytical tools further complicates the interpretation of molecular mechanisms underlying OA progression. OA progression is closely related to mechanical stress, with abnormal mechanical loading leading to chondrocyte homeostasis disruption, triggering inflammation and apoptosis [229]. However, current organoid models struggle to accurately replicate the effects of mechanical stimuli on cell behaviour. While 3D scaffold‐assisted systems can simulate tissue‐matrix interactions, their mechanical properties require further optimisation to match in vivo joint environments [230]. The challenge lies in precisely controlling mechanical forces, including shear stress, compressive strain and hydrostatic pressure, which are crucial for accurately modelling OA pathogenesis. These technological challenges highlight the need for integrating intelligent automation, AI‐driven modelling and advanced biophysical engineering techniques to enhance the biological relevance and clinical applicability of OA organoids in disease modelling and drug discovery (Figure 5).

### 5.1 Software of Intelligent Manufacture

#### 5.1.1 Artificial Intelligence

In the ‘human‐free factory’ OA pathological organoid intelligent manufacturing strategy, AI serves as the core computational engine. AI refers to computational systems that simulate human cognitive functions, including learning, reasoning, perception and decision‐making. Key AI subfields include: Machine learning (ML): Extracts patterns from large‐scale datasets for prediction and classification; Deep learning (DL): Uses multi‐layer neural networks to model complex relationships, widely applied in image recognition and biomedical data processing; Natural language processing (NLP): Analyses and generates scientific text for literature mining and hypothesis generation. These AI technologies are extensively applied in biomedical research, facilitating data analysis, model development and experimental optimisation [231]. Integrating AI into organoid manufacturing enables AI‐assisted construction, analysis and application, significantly enhancing efficiency, precision and reproducibility in organoid production [232]. AI offers a broad range of applications in OA organoid manufacturing, including: Optimisation of culture conditions: AI algorithms analyse large experimental datasets to predict optimal culture parameters, such as temperature, humidity and nutrient concentrations. Additionally, AI can predict cell behaviour and optimise material properties, further enhancing the efficiency and precision of organoid manufacturing. AI‐driven optimisation enables rapid screening of existing construction strategies, refining experimental designs to enhance efficiency, accuracy and quality [233]; Image analysis and feature extraction: AI can rapidly extract multi‐scale features from organoid microscopic images, improving analysis efficiency. For example, convolutional neural networks analyse single‐cell imaging and histological sections, enabling multi‐layer, multi‐perspective structural insights for precise morphological evaluation [234]; Multi‐omics data integration: AI facilitates the fusion of genomics, transcriptomics and proteomics data, supporting streamlined analysis workflows for OA disease modelling [235]; Clinical translation and drug evaluation: In combination with iPSCs, AI enables the generation of patient‐specific in vitro disease models, advancing personalised medicine through high‐throughput drug screening, efficacy prediction and organoid‐based therapeutic assessments [236]. Su et al. introduced the world's first domain‐specific AI model for organoids, Organoid‐GPT (O‐GPT), which explores synergistic effects between intelligent manufacturing and organoid construction. O‐GPT provides state‐of‐the‐art domain knowledge, accurate predictions of organoid construction factors and optimised model‐data analysis pipelines, offering solutions for multi‐scale imaging evaluation and multi‐omics data fusion analysis. This framework establishes a virtual research platform for intelligent manufacturing of pathological OA organoids. Additionally, Bai et al. provided a detailed overview of the fundamental concepts and mechanisms by which AI supports organoid construction, summarising its prospective applications in high‐throughput screening of organoid fabrication strategies, cost‐effective extraction of multi‐scale imaging features, simplified multi‐omics data analysis and precise preclinical evaluation and application [232]. Specifically, AI can drive precise adjustments to the biochemical and biophysical properties of specific hydrogels during organoid fabrication, greatly improving the final organoid formation [237]. Furthermore, AI can predict and identify stem cell differentiation pathways in organoid cultures. Zhu et al. employed deep learning to extract details from large‐scale datasets and developed a deep neural network model for predicting and reliably identifying neural stem cell fate. This model demonstrated high efficiency in recognising differentiated cell types and exhibited remarkable accuracy and robustness across independent test scenarios involving various inducers, including neurotrophic factors, hormones, small‐molecule compounds and nanoparticles [238].

To fully implement AI‐driven automation, three core software systems must be developed for ‘human‐free factory’ OA organoid manufacturing: Automated Culture Optimisation and Real‐Time Monitoring System; Multi‐Modal Image Database and Image Processing System; Multi‐Omics Deep Neural Network System. These three systems function sequentially, interconnecting upstream and downstream processes in a relay‐style workflow, collectively powering the ‘human‐free factory’ intelligent manufacturing system.

#### 5.1.2 Real‐Time Monitoring Feedback System

AI‐driven algorithms can analyse complex datasets and predict critical culture variables, including temperature, oxygen concentration, pH levels, biological aging, nutrient composition and growth factors. By leveraging machine learning models, AI determines the optimal conditions for cell growth, differentiation and organoid formation. This approach enables rapid screening of various biomaterials, growth factors and environmental parameters, facilitating the development of more efficient and effective OA organoid construction strategies. Furthermore, AI‐powered optimisation significantly reduces variability in traditional cell culture processes, thereby enhancing reproducibility and consistency in organoid production. Natsume et al. developed a robotic AI system incorporating a batch Bayesian optimisation algorithm to refine differentiation protocols for iPSCs‐derived retinal pigment epithelial cells, systematically optimising cell culture conditions to achieve superior differentiation outcomes [239]. Similarly, Su et al. utilised machine learning to analyse the spatial structure of hydrogels, predicting cellular behaviour dynamics and assessing the impact of external stimuli (e.g., temperature, chemical agents and enzymatic degradation) on hydrogel performance. This AI‐driven approach enables the identification of optimal hydrogel synthesis strategies for biological applications [240]. In the context of OA organoid manufacturing, AI can be effectively integrated with pathological OA organoid biocharacteristics to systematically optimise: Culture media composition and gradient control; Mechanical stimulation loading patterns; Passaging cycle parameters. By establishing standardised operating procedures, AI ensures precision and reliability in OA organoid culture. Additionally, real‐time monitoring technology is pivotal in culture condition optimisation. By integrating sensors into the culture system, it is possible to dynamically monitor key microenvironmental parameters, such as oxygen concentration, pH levels and nutrient availability. These real‐time data are processed by AI algorithms, which autonomously adjust culture conditions to maintain optimal cellular growth states. This closed‐loop feedback mechanism enhances experimental accuracy and minimises human intervention errors, thereby improving the overall efficiency and reproducibility of OA organoid manufacturing [241].

#### 5.1.3 Image Processing System

In recent years, deep learning technology has been widely applied in the diagnosis and research of OA. To support intelligent manufacturing and data‐driven analysis, it is essential to establish a high‐quality multi‐modal organoid image database. Deep learning‐driven image denoising and 3D reconstruction algorithms can effectively integrate data from multiple imaging modalities, such as X‐ray, CT scans and MRI, forming a comprehensive database containing over 100,000 annotated images. Standardised preprocessing pipelines are implemented to mitigate image blurring and registration artefacts caused by light scattering, ensuring high‐quality datasets for subsequent quantitative analyses [242, 243]. Leveraging this large‐scale multi‐modal image database, an improved U‐Net++‐organoid segmentation algorithm has been developed to precisely quantify morphological parameters, including volume, surface area and sphericity. Furthermore, by integrating Haralick texture features and vascular network topology analysis, critical pathological phenotypes such as synovial hyperplasia and necrotic region distribution in OA can be extracted. Philippe et al. conducted a longitudinal study using 3D MRI data of OA patients, applying deep learning models for MRI analysis to predict disease progression and prognosis [244]. These extracted features not only aid in understanding organoid developmental abnormalities but also provide key insights for early diagnosis and treatment strategies [245, 246]. For implementation, Savani et al. utilised pretrained models, including ResNetV2 and Xception, for data classification and feature recognition. A visualisation platform was developed to integrate growth trajectory tracking and phenotype evolution heatmaps [243]. By combining morphological parameters with functional biomarkers, this approach provides intelligent decision support for high‐throughput experiments.

#### 5.1.4 Multi‐Omics Deep Neural Network System

The development of a multi‐omics deep neural network system aims to analyse the complex relationships between multi‐modal organoid data (genomics, metabolomics, imaging) and culture condition parameters (e.g., growth factors, mechanical stimulation, oxygen levels). By integrating genomics, metabolomics and imaging datasets, this system captures dynamic molecular changes and phenotypic features during organoid development. Genomics data reveal key gene expression regulatory mechanisms. Metabolomics data provide temporal profiles of metabolite concentrations. Imaging data reflect morphological and structural changes in OA organoids [247]. Built on a deep learning framework, this system employs multi‐modal variational encoders to unify diverse datasets into a shared latent representation, with decoders generating predictive outputs. By integrating single‐cell transcriptomic dynamics, metabolite concentration shifts and microscopic imaging features, it establishes a quantitative correlation model between organoid developmental states and culture conditions [248]. This model deciphers the impact of key culture parameters (e.g., growth factor concentration, mechanical stress, oxygen gradients) on organoid differentiation and functional maturation, forming a data‐model‐experiment closed‐loop system. Huang et al. proposed Multi‐Omics Graph Convolutional NETwork, which integrates mRNA, DNA methylation and miRNA data to classify renal carcinoma with higher accuracy than traditional methods [249]. This framework can be extended to OA organoids, optimising graph convolutional network layers and view correlation discovery networks to analyse multi‐omics responses to culture conditions. To address the small‐sample data limitation, this system incorporates a reinforcement learning‐based culture condition optimisation algorithm. RL mimics human decision‐making processes, continuously refining strategies through trial and error. In OA organoid culture, RL algorithms can simulate dynamic experimental conditions, predicting optimal culture parameter combinations. Additionally, by integrating generative adversarial networks (GANs), high‐quality synthetic datasets can be generated to compensate for real‐world data scarcity. GANs train generators and discriminators, producing synthetic data distributions that closely resemble real biological datasets, thereby enhancing model generalisability [250, 251]. Carlo et al. proposed a bio‐manufacturing optimisation method combining reinforcement learning and simulation, in which environmental state observations, action generation and reward collection iteratively refine optimal biomanufacturing strategies, ultimately generating optimised target sequences [252].

### 5.2 Hardware of Intelligent Manufacture

#### 5.2.1 Microfluidic Equipment

In the ‘human‐free factory’ intelligent manufacture, the microfluidic microporous array chip can be constructed in a modular manner, allowing for precise and dynamic design of the stem cell culture environment. This enables comprehensive simulation of the cellular components of the inflammatory microenvironment, inflammatory factor composition and immune factors, ultimately creating organoids that accurately reflect the pathological characteristics of OA. Its core functions and advantages include: *Cell Capture and Aggregation*: The precise microwell array design enables effective cell capture and aggregation, establishing a stable physical foundation for organoid formation. For instance, hexagonally arranged microchambers (≥ 1000 wells per chip) optimise cell distribution. Fibronectin‐coated microwell surfaces enhance cell adhesion, thereby improving culture efficiency [253]; *Organoid Size Control and Standardisation*: Microfluidic chips simulate fluid shear forces and optimise microwell spacing and bottom micro‐pillar structures, ensuring high uniformity in organoid size. This design minimises batch‐to‐batch variability, providing a reliable framework for standardised OA organoid manufacturing [254]; *Integration of Miniature Biosensor Arrays*: The microfluidic chip is embedded with miniaturised biosensor arrays, which, when combined with the real‐time monitoring and feedback system, enable continuous tracking of key culture parameters (e.g., oxygen concentration, temperature). These sensors dynamically regulate culture conditions, ensuring precise control over organoid development. This real‐time sensor integration enhances mechanistic studies of OA and provides valuable feedback for disease modelling [255]; Development of Modular Culture Platforms: The automated organoid culture platform, based on microwell array technology, supports high‐throughput, parallelised and standardised organoid production. Each module contains six independent microfluidic chips, equipped with miniature heating units and gas exchange membranes, enabling simultaneous multi‐batch culture. This modular approach improves experimental efficiency while significantly reducing production costs [254].

#### 5.2.2 Automated Liquid Handling Equipment

With the increasing demand for biomedical research and clinical applications, automated liquid handling equipment is pivotal in the scalable production and precise management of organoids. These equipment integrate multiple functions, including cell seeding, passaging, cryopreservation, media exchange and real‐time monitoring, enabling fully automated workflows from cell culture to organoid generation. By evaluating batch‐to‐batch variability coefficients, these equipment validate product stability and enhance the efficiency of large‐scale organoid manufacturing. Schimitt et al. proposed an automated organoid culture method known as AutoCRAT, which enables large‐scale automated production of MSCs, chondrocytes and extracellular vesicles derived from induced iPSCs [256]. The core components of the automated liquid handling equipment include: High‐Precision Robotic Arms: The equipment employs high‐precision robotic arms to ensure accurate and reproducible cell seeding and handling. These robotic arms precisely execute cell positioning and manipulations, minimising human error and improving experimental reproducibility. Ioannis et al. developed a robotics‐based automation strategy for media exchange and imaging of cartilaginous microtissues cultured in static microwell platforms, demonstrating the efficiency and consistency of robotic‐assisted handling [257]; High‐Accuracy Syringe Pumps: The equipment is equipped with syringe pumps with flow rate errors below 1%, enabling precise control over liquid dispensing. This precision is particularly critical for media replacement and cryopreservation solutions, ensuring stable and reproducible culture conditions [258]; Miniaturised Cryopreservation Module: The cryopreservation module features a rapid cooling rate of 10°C/min, designed for gradient cooling with dimethyl sulfoxide (DMSO) during cell cryopreservation. This function enhances cryopreservation efficiency while minimising cellular damage during the freezing process [256].

#### 5.2.3 Multi‐Parameter Sensing Equipment

In the ‘human‐free factory’ intelligent manufacturing strategy, the multi‐parameter sensing equipment is a core technology enabling real‐time monitoring and intelligent regulation of the organoid microenvironment. This equipment integrates miniaturised sensors for pH, oxygen partial pressure, metabolites (e.g., lactate/glucose) and mechanical stress detection, constructing a multi‐dimensional dynamic data acquisition platform for real‐time environmental monitoring. These sensors operate at millisecond‐level temporal resolution, continuously tracking key biophysical and biochemical parameters of organoids. Key Components of the Multi‐Parameter Sensing Equipment include: pH and Oxygen Partial Pressure Sensors: The detection of pH and oxygen levels primarily relies on fluorescent fibre‐optic sensor technology. Sensor probes are coated with Ru(dpp)_3_^+^ oxygen‐sensitive films, which emit fluorescence signals in response to oxygen concentration fluctuations, enabling real‐time oxygen monitoring [259]. Additionally, pH‐sensitive visualisation sensors based on metal–organic frameworks and covalent organic frameworks have been extensively studied. These sensors are designed to enhance pH measurement accuracy and emit multi‐wavelength fluorescence signals, improving detection sensitivity [260]; Metabolite Monitoring: The monitoring of metabolites (e.g., lactate and glucose) is achieved using microfluidic electrochemical chips. These chips incorporate glucose oxidase and lactate dehydrogenase‐modified electrodes, allowing for precise metabolite concentration detection. This enables researchers to assess the metabolic state of organoids under specific culture conditions [261]; Mechanical Stress Sensors: The equipment integrates a flexible piezoresistive sensor array, designed to detect compression stress within the organoid microenvironment. These sensors feature a measurement range of 0–20 kPa and a response time of less than 10 milliseconds, enabling real‐time tracking of mechanical forces acting on the organoid. This is critical for simulating the mechanobiological stimuli present in the pathophysiological environment of OA [262]. Building upon the multi‐parameter sensing equipment, researchers have further developed an intelligent regulation equipment. This equipment utilises a microfluidic valve array to dynamically adjust: Culture medium perfusion rates; Growth factor release gradients; Mechanical stress loading patterns. By comparing static and dynamic culture equipments, studies have demonstrated that dynamic regulation significantly enhances organoid maturation and functional properties. This equipment serves as a technological foundation for simulating in vivo pathological microenvironments, advancing next‐generation organoid‐based disease modelling [259].

#### 5.2.4 Imaging Equipment

In recent years, with the rapid advancement of biomedical imaging technologies, multi‐photon microscopy (MPM) and light‐sheet fluorescence microscopy (LSFM) have demonstrated tremendous potential in organoid research due to their unique imaging advantages. To overcome the depth and resolution limitations of traditional imaging techniques, a combined multi‐photon confocal microscopy and light‐sheet imaging equipment has been developed, enabling cross‐scale simultaneous capture and measurement of organoid 3D structures and subcellular dynamics. This equipment integrates the strengths of both MPM and LSFM and allows rapid switching between imaging modalities via a customised sample stage. Multi‐photon confocal microscopy utilises laser excitation wavelengths of 690–1300 nm, achieving a Z‐axis resolution of up to 10 μm. Light‐sheet fluorescence microscopy employs dual‐laser excitation at 488/561 nm, providing an XY resolution as high as 0.5 μm. This design significantly enhances imaging depth, reduces phototoxicity and enables high‐resolution imaging of deep tissues [263]. Additionally, by incorporating a piezoelectric ceramic‐driven 3D sample translation equipment, this setup supports long‐term continuous observation and automated multi‐field stitching, ensuring reliable imaging for over 24 h in studies involving cells, organoids and live tissues. Steventon et al. successfully applied this continuous observation and automatic stitching equipment in long‐term zebrafish studies [264]. To further improve deep‐tissue imaging quality, an adaptive optics module has been introduced into the optical path of the light‐sheet microscope. This module compensates for aberrations caused by light scattering within organoid structures, ensuring clear and accurate deep imaging. Such optimisation not only enhances imaging quality but also provides critical support for analysing complex biological structures. Luke et al. demonstrated this by employing an adaptive optics module for imaging mitochondria through bone in live mice using two‐photon fluorescence microscopy [265].

### 5.3 Hardware‐Software Interaction of Intelligent Manufacture

The complete process of ‘human‐free factory’ OA organoid manufacturing is realised through the seamless integration of three major software systems, which serve as the stable core driving the hardware components to perform their designated functions. Essentially, this process entails the comprehensive interconnection between software and hardware equipment, where both complement each other to achieve fully automated operations. The intelligent manufacturing process of the ‘human‐free factory’ utilises microfluidic microporous array chips, automated liquid handling equipment and intelligent establishment of culture conditions to enable high‐throughput automated culture of osteoarthritis organoids. Simultaneously, a multi‐parameter sensing equipment and a real‐time monitoring feedback system ensure real‐time detection and dynamic regulation of the culture environment, facilitating a ‘serial’ organoid culture approach. To process the massive image data generated during automated culture, the ‘human‐free factory’ intelligent manufacturing system integrates a multi‐photon confocal microscopy and light‐sheet imaging equipment to achieve multi‐scale, high‐resolution imaging. Additionally, a multi‐modal image database and an image processing equipment are established to enable image feature extraction and analysis. Beyond multi‐modal imaging data, the organoid culture process also generates extensive multi‐omics data, including genomics, metabolomics and radiomics. The ‘human‐free factory’ system constructs deep neural networks for multi‐omics data integration, develops fusion algorithms and models and completes model validation and optimisation of culture conditions. Through continuous refinement of technical details and addressing technological shortcomings, the above ‘human‐free factory’ manufacturing process aims to facilitate clinical translation, providing a novel paradigm for the pathological organoid manufacturing of osteoarthritis and related diseases. This advancement is expected to further drive disease mechanism research, drug screening and the steady development of precision medicine.

## 6 Challenges and Prospects

The intelligent manufacturing of OA organoids faces three core challenges: pathological microenvironment replication, smart technology integration and scalable production. Current models struggle to dynamically simulate multi‐tissue interactions (e.g., chondrocytes, osteoblasts, immune cells) critical to OA progression, particularly the dynamic interplay of inflammatory cascades and cartilage metabolic imbalance, while static culture systems fail to mimic the joint cavity's mechanical stress microenvironment, demanding the development of dynamic mechanical loading technologies and advanced biomaterials [266, 267]. In AI integration, limitations persist in multi‐omics data analysis, with algorithmic models prone to information loss or overfitting, compounded by the ‘black‐box’ nature hindering mechanistic interpretation. Real‐time adaptive control remains constrained by offline data dependency, requiring edge computing and embedded AI chips for millisecond‐level closed‐loop regulation [268, 269]. Scaling production confronts organoid heterogeneity‐induced batch variability (cell distribution, matrix composition), inconsistent multi‐site reproducibility due to non‐standardised culture parameters (e.g., oxygen gradients) and a lack of GMP standards for intelligent biomanufacturing [239, 270]. Regulatory ambiguities—including ambiguous classification of AI‐integrated organoids (biologics vs. medical devices), undefined quality metrics for dynamic cultivation and fragmented international standards for machine learning‐driven production—further impede industrial translation. With the rapid advancements in organoid culture techniques and intelligent technologies, these challenges are expected to be effectively addressed in the near future.

**FIGURE 6:**
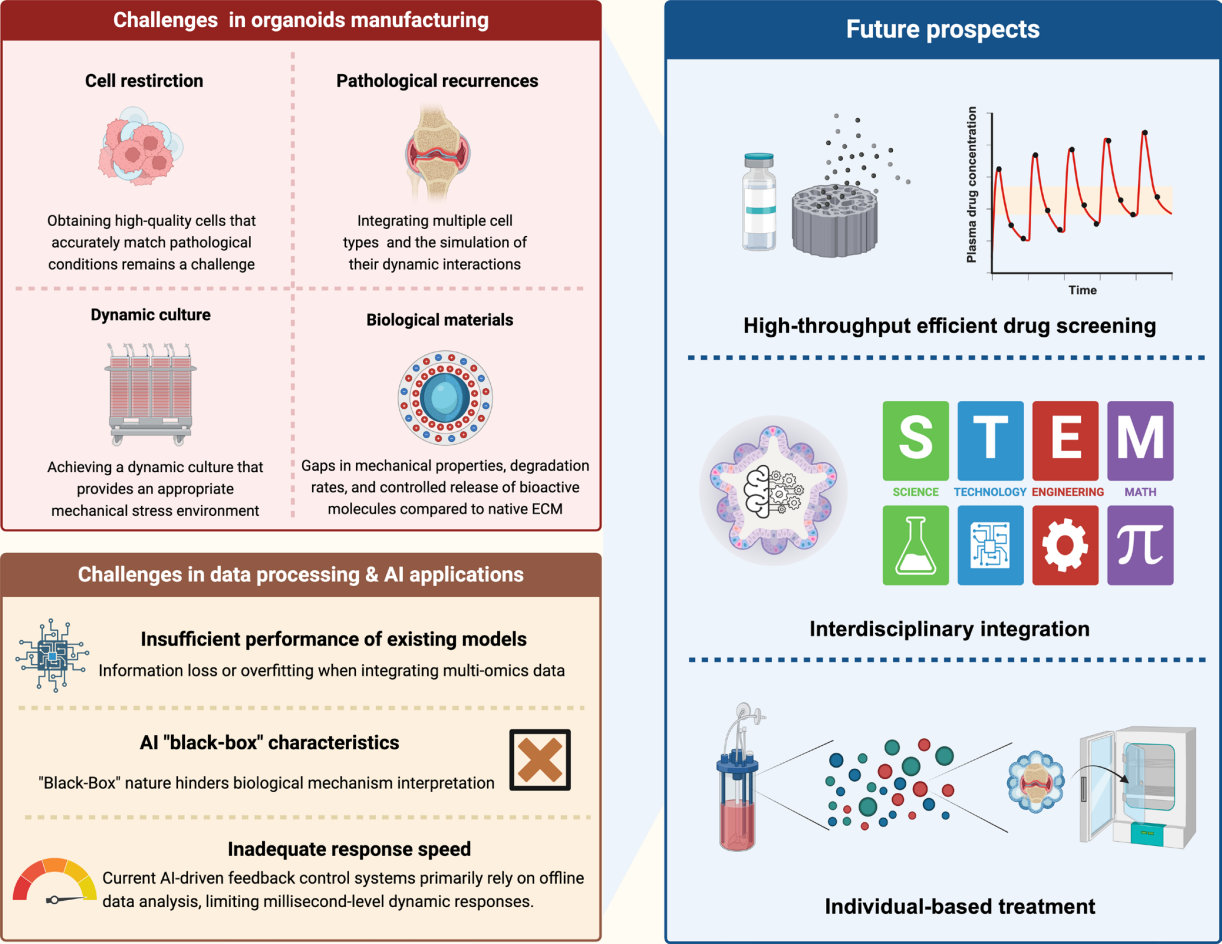
Challenges and future outlook in osteoarthritis organoid research. Summarising current obstacles—including issues related to cell sourcing, dynamic culture, material selection and data processing—this figure also highlights potential avenues for high‐throughput drug screening, interdisciplinary integration and personalised therapy. Created with BioRender.com.

Overcoming current technological bottlenecks requires synergistic innovations across biomaterials science, bioengineering and AI technologies: Developing intelligent responsive hydrogels to simulate dynamic mechanical microenvironments. Leveraging organ‐on‐a‐chip technology to achieve multi‐tissue coupling, thereby enhancing organoid functionality [271]. Accelerating the adoption of high‐throughput intelligent manufacturing platforms to reduce organoid production costs. By integrating organoid models into high‐throughput drug screening, therapeutic validation and prospective clinical trials, organoid technology can be effectively translated from fundamental research to personalised medicine. By integrating patient genomic data into the construction of individualised organoid models, researchers can more accurately predict drug responses and optimise therapeutic strategies, thereby advancing the development of personalised medicine. For example, constructing genotypic organoids based on GWAS‐identified susceptibility genes could provide a precision screening platform for targeted therapies (e.g., RNA interference, gene editing). Furthermore, establishing a globally coordinated ethical review framework is necessary to define the boundaries of organoid technology in research and clinical applications. Simultaneously, public science communication efforts should be strengthened to enhance societal acceptance of this emerging technology (Figure 6).

## Author Contributions

**Xukun Lyu:** writing – original draft, methodology, investigation, formal analysis, data curation, conceptualisation. **Jian Wang:** writing – review and editing, supervision, resources, formal analysis, conceptualisation. **Jiacan Su:** writing – review and editing, supervision, project administration, resources, funding acquisition, conceptualisation.

## Conflicts of Interest

The authors declare no conflicts of interest.

## Data Availability

Data sharing not applicable to this article as no datasets were generated or analysed during the current study.
